# Resting-State Functional Connectivity Profile of Insular Subregions

**DOI:** 10.3390/brainsci14080742

**Published:** 2024-07-25

**Authors:** Jimmy Ghaziri, Phillip Fei, Alan Tucholka, Sami Obaid, Olivier Boucher, Isabelle Rouleau, Dang K. Nguyen

**Affiliations:** 1 Département de Psychologie, Université du Québec à Montréal, Montréal, QC H2X 3P2, Canada; 2 Centre de Recherche du Centre Hospitalier de l’Université de Montréal (CRCHUM), Montréal, QC H2X 0A9, Canada; 3 Faculté de Médecine et des Sciences de la Santé, Université de Sherbrooke, Chicoutimi, QC J4L 1C9, Canada; 4 BarcelonaBeta Brain Research Center, Pasqual Maragall Foundation, 08005 Barcelona, Spain; 5 Pixyl Medical, 38700 Grenoble, France; 6 Service de Neurologie, Centre Hospitalier de l’Université de Montréal (CHUM), Montréal, QC H2X 0C1, Canada

**Keywords:** insula, insular, connectivity, resting state, rsfMRI, functional, network, salience

## Abstract

The insula is often considered the fifth lobe of the brain and is increasingly recognized as one of the most connected regions in the brain, with widespread connections to cortical and subcortical structures. As a follow-up to our previous tractography work, we investigated the resting-state functional connectivity (rsFC) profiles of insular subregions and assessed their concordance with structural connectivity. We used the CONN toolbox to analyze the rsFC of the same 19 insular regions of interest (ROIs) we used in our prior tractography work and regrouped them into six subregions based on their connectivity pattern similarity. Our analysis of 50 healthy participants confirms the known broad connectivity of the insula and shows novel and specific whole-brain and intra-connectivity patterns of insular subregions. By examining such subregions, our findings provide a more detailed pattern of connectivity than prior studies that may prove useful for comparison between patients.

## 1. Introduction

The insula, often referred to as the fifth lobe of the brain, is enclosed beneath the lateral fissure by the overlying frontal, parietal, and temporal opercula and is divided into two major areas by the central insular sulcus [1,2]. The rostral portion is composed of three short insular gyri (anterior, middle, and posterior) while the caudal region comprises two long insular gyri (anterior and posterior). They all converge inferiorly in an oblique fashion at the apex and form a triangular fan-shaped structure [2,3,4].

Over the past decade, advances in diffusion-weighted imaging have significantly enhanced our understanding of the structural connectivity of the insula. Studies, including ours, have revealed a rostro-caudal connectivity matrix linking the insula to most of the brain regions. The rostral insula primarily connects with the orbitofrontal, superior temporal, and anterior cingulate gyri, whereas the caudal insula mainly projects to the pre- and postcentral gyri, posterior parietal cortex, and occipital cortex. Additionally, the insula has extensive connections with the limbic and subcortical regions, including the thalamus, hippocampus, amygdala, nucleus accumbens, caudate nuclei, and globus pallidus [5,6,7,8,9,10].

Resting-state fMRI (rs-fMRI) allows for the investigation of synchronous temporal signal activations defined by statistical dependency (e.g., correlation, coherence, etc.) in distinct brain regions independent of direct structural connections [11,12,13]. Most functional studies of the insula have utilized divisions based on anatomical features, intrinsic functional connectivity, structural covariance, and task-based coactivation. These studies generally support a bipartite division, revealing an antero-posterior connectivity pattern that mirrors structural and cytoarchitectural findings [14,15,16,17,18,19,20,21]. Overall, the anterior portion was found to be mainly correlated with areas involved in cognitive–affective processes, which include the frontal, temporal, and parietal lobes, the cingulate gyrus, basal ganglia, thalamus, and amygdala. The posterior insula was primarily found to be correlated with sensorimotor areas, including the frontal, temporal, and parietal opercula, as well as the cingulate cortex, primary motor cortex (M1), primary sensory cortex (S1), occipital lobe, and thalamus. Tripartite studies have further divided the anterior insula into dorsal and ventral areas participating, respectively, in cognitive and affective processes [16,18,20].

Meta-analyses and network-based clustering approaches have provided further insights into the functional subdivisions of the insula. For instance, Kurth et al. [19] identified four major functional insular regions, each associated with different task categories, such as sensorimotor, olfacto-gustatory, socio-emotional, and cognitive tasks. This seminal work used activation likelihood estimation meta-analyses of 1768 functional neuroimaging experiments to highlight the specific roles of these regions. Similarly, Kelly et al. [17] demonstrated the convergence of structural and functional covariances, highlighting the complementary nature of these measures in understanding intrinsic network organizations. Their meta-analysis of 355 participants showed that insular clusters between two and nine subregions had the highest consistency and stability across the modalities. They also showed a high degree of overlap between the clusters (>4) and their associated activation tasks. This work underscored the high degree of overlap between the clusters and their associated activation tasks. Another meta-analysis by Uddin et al. [20], analyzing the coactivation profiles of insular subdivisions based on a tripartite division of the insula (dorsal anterior, ventral anterior, and posterior), examined 32 task domains across 4393 studies. They found that the posterior insula coactivated with somatomotor regions, the ventral anterior insula coactivated with inferior frontal regions extending into the orbitofrontal cortex and anterior temporal lobe, and the dorsal anterior insula coactivated with the lateral prefrontal cortex, superior parietal cortex, and midcingulate gyrus. Thus, they showed that the dorsal areas were more related to cognitive tasks, the ventral anterior areas to emotional tasks, and the posterior areas to somatomotor tasks.

In 2016, Glasser et al. [22] conducted a seminal study that advanced our understanding of brain parcellation, including the division of the insula. Their work utilized data from the Human Connectome Project (HCP) to create a highly detailed parcellation of the cerebral cortex. This study integrated multimodal neuroimaging data, including structural MRI, functional MRI (task-based and resting-state), and diffusion-weighted imaging, to define cortical areas with unprecedented precision. For the insula, Glasser et al. delineated several distinct subregions, highlighting the complex and multifaceted nature of this brain area [22].

Few studies have directly investigated the rsFC of the insula. These are based on a small number of insular subregions (two and three) and participants (*N* = 17–20) [14,15,16]. Other studies [17,22,23,24] which have found more (>4) insular functional subdivisions (using postmortem MRI and multimodal imaging) do not report their connectivity profile. Hence, the objective of the current work was to explore the functional connectivity of the insula on a larger number of participants and by using a more refined parcellation of the insula based on our previous structural work [9].

The primary aim of this work is to delineate the rsFC of insular subregions with greater precision and in a larger population than previously studied. This study builds on our previous structural work [9], which provided a detailed parcellation of the insula based on diffusion-weighted imaging data. By leveraging advanced rsfMRI techniques and a larger cohort, we aim to map the functional connectivity of the insula’s subregions more comprehensively. By elucidating these connections, we hope to contribute to a more detailed and accurate model of brain network organization, which could have significant implications for both basic neuroscience and clinical applications. For instance, improved knowledge of insular connectivity could aid in understanding and treating conditions such as anxiety, depression, and chronic pain, which have been linked to dysregulations in insular function [25,26].

## 2. Materials and Methods

Fifty healthy right-handed subjects between the ages of 20 and 40 years (M = 29.98, SD = 5.64; 28 women (M = 30.40, SD = 5.60); 22 men (M = 29.35, SD = 5.79)) with no history of neurological or psychiatric disorders were recruited. All participants gave informed written consent for procedures. The study was approved by the Centre Hospitalier de l’Université de Montréal (CHUM) ethics committee, per the latest revision of the Declaration of Helsinki.

MRI data were acquired on a 3T Achieva scanner (Philips, Amsterdam, The Netherlands). The rsfMRI images were acquired with a single-shot spin-echo echo-planar pulse sequence (TR = 2000 ms; TE = 30 ms; flip angle = 90°; slices = 36; field of view = 240 mm; matrix = 80 × 80; voxel resolution = 3.00 × 3.00 × 3.00 mm; echo-planar imaging direction bandwidth = 2185.4 Hz; 32-channel head coil; SENSE acceleration factor = 1). Additionally, T1-weighted images were acquired using 3D T1 gradient echo (scan time = 5.56 min; TR = 2000 ms; TE = 30 ms; flip angle = 90°; slices = 170; voxel size = 1 × 1 × 1 mm; field of view = 240 × 240 mm).

**Figure 1:**
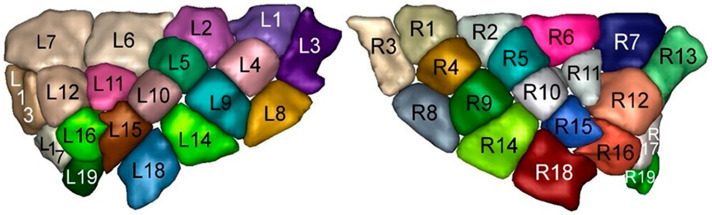
Nineteen insular subregions in the left and right hemispheres.

**Figure 2:**
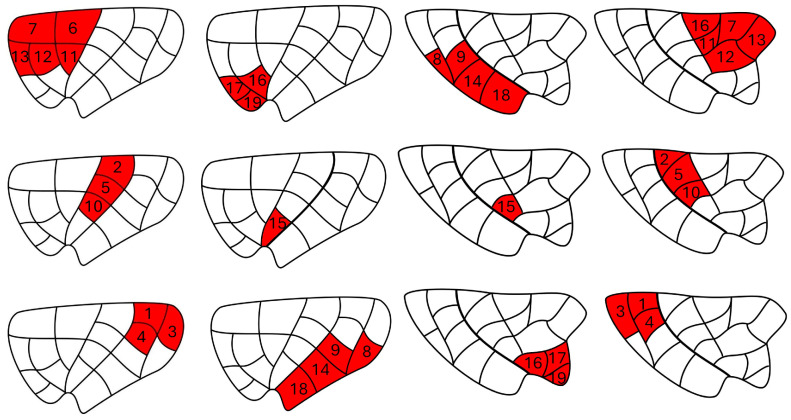
Six insular subregions in the left and right hemispheres.

Insular regions of interest were determined from our previous structural diffusion tractography work, which consists of 19 subregions in each hemisphere (see [9] for details on the parcellation; Figure 1). They were registered from T1-weighted space to functional space using ANTs registration tool (https://stnava.github.io/ANTs/; last accessed on 4 September 2021) and visually checked by three investigators (J.G., PhD student in neuropsychology; A.T., neuroimaging expert; and D.K.N., neurologist specializing in insular epilepsy). The 19 insula ROIs were consolidated into six primary ROIs based on the similarity of their functional connectivity patterns. To facilitate reading and comparison with previous studies [9], we found it simpler to merge subregions that exhibited similar statistical correlation maps (connectivity profiles), resulting in six distinct subregions (Table 1, Figure 2).

Preprocessing steps and analyses were carried out using the CONN functional connectivity toolbox (version 18.a; www.nitrc.org/projects/conn; last accessed on 18 June 2018) [27] in combination with SPM12 software (http://www.fil.ion.ucl.ac.uk/spm/software/spm12/; last accessed 18 June 2018). The pipeline consists of slice-timing correction, realignment (motion corrected), unwarping resting-state functional raw data and then coregistration to every participant’s T1-weighted structural image. Images were then normalized to the Montreal Neurological Institute (MNI) standardized coordinate space, spatially smoothed to 8mm full-width at maximum half, and resliced to 2 × 2 × 2 mm voxels [28] using SPM’s segmentation and normalization algorithm [29,30].

Initially, functional data were realigned using SPM’s realign and unwarp procedure [31]. All scans were coregistered to a reference image using a least squares method and a 6-parameter (rigid body) transformation [32]. The scans were resampled via b-spline interpolation to correct for motion and magnetic susceptibility interactions. Temporal misalignment between slices was addressed using SPM’s slice-timing correction (STC) procedure [33,34].

Motion artifact detection and rejection (scrubbing) were performed using the Artifact Detection Tool (ART; http://www.nitrc.org/projects/artifact_detect/). This tool regresses out scans if the head displacement in all three directions is higher than 0.5 mm from the previous frame, if the rotational displacement is higher than 0.02 radians from the previous frame, or if the global mean intensity in the image was higher than three standard deviations (SDs) from the mean image intensity for the entire resting scan [35]. Outliers in the global mean signal intensity and motions were then added to the six rotation/translation movement parameters as nuisance covariates [36].

Noise reduction was performed using the recommended parameters [37] of the anatomical component-based noise correction method (aCompCor) [38,39]. The specificity and sensitivity of this tool for positive correlations are higher than those of a global regression method, while it does not deserve artifactual anticorrelations. BOLD signals obtained from the white matter and CSF segmented maps are extracted from the functional volumes by using principal component analysis and then used as confounds [40]. These confounds consist of motion parameters [35], session effects, linear trends, and outlier scans of each subject’s eroded segmentation mask [41]. After regressing out the noise from these ROIs, the resulting BOLD time series were band-pass filtered (0.008–0.09 Hz) to reduce low-frequency drifts and noise effects [42,43,44].

We employed seed-based analysis (seed-to-voxel) to explore the resting-state functional connectivity of insular subregions. This approach involves selecting all 19 subregions, followed by the 6 main merged insular subregions as seeds, from which we assess functional connectivity with the entire brain. By correlating the time series of the seed regions with all other voxels, we generate voxel-wise connectivity maps that reveal the spatial distribution of functional connections associated with each seed. This method is particularly advantageous for exploratory studies, as it allows for the identification of widespread connectivity patterns that may not be captured through predefined ROI-to-ROI comparisons [45,46]. We believe that this seed-based approach facilitates the identification of connectivity patterns specific to the insular subregions compared to an approach based on ROI-to-ROI. Therefore, Residual BOLD time series from insular ROIs were employed to compute the temporal Pearson’s correlation coefficients between these seed ROI time series and the time series of all other brain voxels. Motion parameters and noise ROIs were used as within-subject covariates. The resulting correlation maps were transformed into normally distributed scores using Fisher’s r-to-z transformation. This generated seed-to-voxel connectivity maps for each subject, which were then used for second-level analyses [47].

For within-group analysis, we conducted second-level analyses using cluster-level inferences based on Gaussian Random Field theory [48,49]. Statistical parametric maps of T values were estimated using the general linear model (GLM) [50] controlling for multiple comparisons across the entire brain volume. Each voxel’s first-level connectivity measures were treated as dependent variables in separate GLMs. Results were thresholded with a combination of a cluster-forming threshold of *p* < 0.001 at the voxel level and a familywise corrected p-FDR < 0.001 at the cluster-size level [51] ensuring that any detected clusters of connectivity were statistically significant. Regions were identified using the Harvard–Oxford atlas [52,53,54,55] and visualized on an MNI template.

## 3. Results

### 3.1. Seed-to-Voxel

The 19 insular subregion connectivity profiles are available in the Supplementary Materials (Figures S1 and S2, and Table S1) along with their cluster size and peak coordinates (Table S2). Descriptive figures of the ROI-to-ROI results are also available in the Supplementary Materials (Figures S3–S12).

All the seed-to-voxel results shown are based on a primary height threshold (*p* < 0.001) followed by an FDR corrected at the cluster-level (*p* < 0.001) threshold. The color bar of each figure represents the highest t-value for the hot colors (positive correlation) and the lowest t-value for the cold colors (negative correlation).

**Figure 3:**
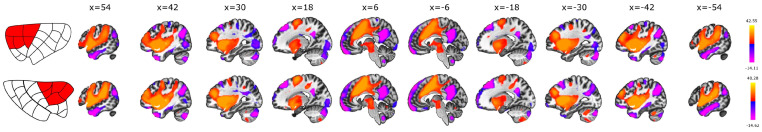
Functional connectivity of the left and right dorsal anterior insula (dAI).

The dorsal anterior insula (dAI; Figure 3) was functionally correlated with the frontal, superior temporal, parietal, and cingulate regions, as well as with the putamen, globus pallidum, amygdala, and left nucleus accumbens. Its strongest correlations were with the frontal opercula, anterior cingulate cortex (ACC), SMA, temporal operculum, orbitofrontal cortex, planum temporale, and supramarginal gyrus (SMG). The dAI is anticorrelated with the middle and right inferior temporal gyrus, the precuneus, the superior lateral occipital cortex, and the right inferior lateral occipital cortex. When comparing the hemispheric differences, the left-most anterior portion of the dAI is mainly correlated to the ventral frontal areas such as the orbitofrontal cortex, while the left most-dorsal portion of the dAI was mainly correlated with the frontal and temporal opercula, SMA, SMG, and ACC. The left-most postero-ventral portion of the dAI was mainly correlated with the ACC, frontal operculum, planum polare, Heschl’s gyrus, and the SMA. However, the right dAI showed a preferential correlation only to the ipsilateral orbitofrontal cortex.

**Figure 4:**
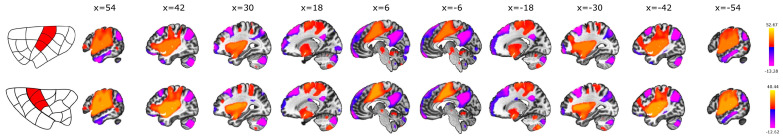
Functional connectivity of the left and right dorsal middle insula (dMI).

The dorsal middle insula (dMI; Figure 4) was essentially correlated with regions similar to the dAI but was connected to more parietal areas and with no connections to the nucleus accumbens. The main functional correlations of the dMI were with the temporal operculum, Heschl’s gyrus, planum polare, planum temporale, parietal operculum, SMA, supramarginal gyrus, precentral gyrus, and anterior cingulate cortex. The dMI was functionally anticorrelated with the frontal pole, left superior frontal gyrus, middle frontal gyrus, middle temporal gyrus, left angular gyrus, precuneus, superior lateral occipital cortex, and posterior cingulate cortex. Individually, every ROI had its highest correlation with the temporal operculum. The most dorsal portion of the dMI showed a significant functional correlation with the precentral gyrus, postcentral gyrus, and SMA, while the most ventral portion of the dMI was mainly correlated with the ACC and SMA in addition to auditory areas. A similar pattern of connectivity can be seen for the right dMI ROIs.

**Figure 5:**
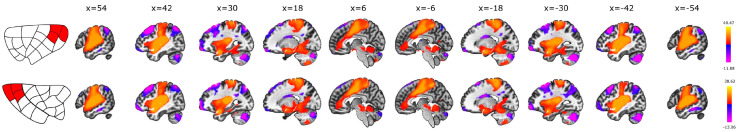
Functional connectivity of the left and right dorsal posterior insula (dPI).

The dorsal posterior insula (dPI; Figure 5) was positively correlated with regions similar to the dAI but with additional correlations with the parahippocampal gyrus and hippocampus. However, its main correlations were with peri-auditory cortical areas, temporal and parietal opercula as well as the pre- and postcentral gyri. The dPI was anticorrelated with the frontal pole, middle frontal gyrus, angular gyrus, and superior lateral occipital cortex. Unique functional connections, compared to all other insular subregions, were found between the left dPI and contralateral temporal occipital fusiform cortex as well as between the right dPI and the supracalcarine cortex. It was also the only insular subregion that had no correlations with the right orbitofrontal cortex. Overall, the highest correlations were seen with the auditory areas followed by the frontal, parietal, and superior temporal regions for both hemispheres.

**Figure 6:**
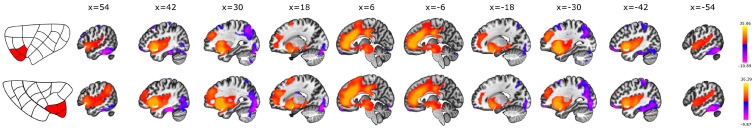
Functional connectivity of the left and right ventral anterior insula (vAI).

The ventral anterior insula (vAI; Figure 6) was correlated with the paracingulate gyrus, ACC, orbitofrontal cortex, and frontal opercula. The left vAI was also correlated with the medial frontal cortex, posterior cingulate cortex, amygdala, hippocampus, and nucleus accumbens. The right vAI was the only subregion functionally connected to the right frontal pole, right posterior middle temporal gyrus, right anterior inferior temporal gyrus, and right angular gyrus. It is also the only region that was not connected with the precentral gyrus, right posterior superior temporal gyrus, and right anterior SMG. Otherwise, it was mainly correlated with the ACC, ipsilateral orbitofrontal cortex, frontal operculum, superior frontal gyrus, amygdala, contralateral nucleus accumbens, and bilateral paracingulate cortex. Finally, the vAI was anticorrelated with the inferior temporal, posterior fusiform, and superior lateral occipital areas.

**Figure 7:**
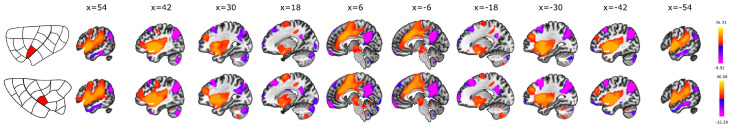
Functional connectivity of the left and right ventral middle insula (vMI).

The ventral middle insula (vMI; Figure 7) was mainly correlated with the ACC and peri-insular opercula. It was anticorrelated with the middle frontal gyrus, posterior inferior temporal gyrus, left angular gyrus, precuneus, and superior lateral occipital cortex.

**Figure 8:**
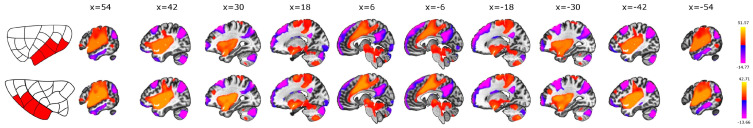
Functional connectivity of the left and right ventral posterior insula (vPI).

The ventral posterior insula (vPI; Figure 8) was mainly correlated with the temporal and parietal opercula, peri-auditory areas, ACC, temporal pole, superior temporal gyrus, supramarginal gyrus, pre-, and postcentral gyrus, and SMA. It was anticorrelated with the frontal pole, left superior frontal gyrus, middle frontal gyrus, inferior and middle temporal areas, left angular gyrus, and superior lateral occipital cortex. The left vPI had unique functional connections with the anterior temporal fusiform cortex. Overall, both hemispheres show a similar pattern of connectivity.

### 3.2. Intrinsic Intra-Insular Connectivity

Intra-insular connectivity correlations were also observed. Here, we compare the intrinsic functional connectivity between the anterior and posterior subregions (Figure 4), dorsal and ventral subregions (Figure 5), and both hemispheres (Figure 6).

### 3.3. Anterior vs. Posterior

**Figure 9:**
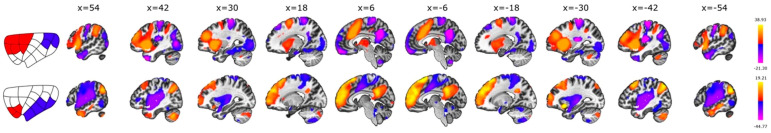
Left and right dorsal anterior (dAI) vs. dorsal posterior (dPI) functional connectivity of the left and right insular subregions.

The dAI was found to be more functionally connected to the frontal regions, the planum polare, SMG, paracingulate gyrus, ACC, right caudate nucleus, putamen, globus pallidum, and right nucleus accumbens when compared with the dPI. However, the dPI connected strongly with the middle temporal gyrus, right anterior inferior frontal gyrus, temporal fusiform cortex, postcentral gyrus, precuneus, occipital areas, and hippocampus (Figure 9).

**Figure 10:**
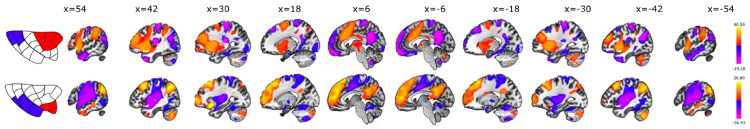
Left and right ventral anterior (vAI) vs. ventral posterior (vPI) functional connectivity of the left and right insular subregions.

The vAI also appeared to be more functionally connected with the frontal regions when compared with the vPI. Additionally, it had stronger connections in the posterior middle temporal gyrus, anterior inferior temporal gyrus, right angular gyrus, precuneus, paracingulate cortex, and caudate nucleus. The vPI was functionally connected more with the precentral gyrus, SMA, superior temporal areas, postcentral gyrus, superior parietal cortex, SMG, parietal operculum, anterior cingulate cortex, and amygdala (Figure 10).

### 3.4. Dorsal vs. Ventral

**Figure 11:**
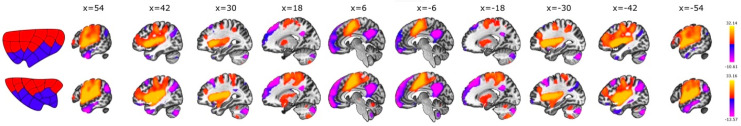
Dorsal vs. ventral functional connectivity of insular subregions (top: left hemisphere; bottom: right hemisphere).

The general connectivity pattern observed when comparing the dorsal and ventral insular regions (Figure 11), showed that the dorsal insula had more functional connections to the inferior frontal areas, precentral gyrus, peri-insular opercula, auditory and peri-auditory regions, postcentral gyrus, superior parietal cortex, supramarginal gyrus, anterior cingulate cortex, and putamen. The ventral portion was more connected to the frontal pole, medial frontal cortex, middle temporal gyrus, anterior inferior temporal gyrus, precuneus, occipital regions, and posterior cingulate cortex.

When comparing the dAI to its ventral counterpart, the dAI was more connected to the posterior inferior frontal regions, the precentral gyrus, SMA, orbitofrontal regions, peri-insular opercula, auditory and peri-auditory regions, superior parietal cortex, SMG, anterior cingulate cortex, putamen, and globus pallidum. The vAI was found to be more connected to the frontal medial cortex, middle temporal gyrus, anterior inferior frontal gyrus, left angular gyrus, precuneus, superior lateral occipital cortex, sub-calcarine cortex, posterior cingulate cortex, and hippocampus.

The dMI was more correlated with the right pars opercularis, precentral gyrus, SMA, peri-insular opercula, superior temporal gyrus, auditory and peri-auditory regions, postcentral gyrus, superior parietal lobule, SMG, anterior cingulate cortex, putamen, and amygdala. The vMI was more connected with the frontal pole, middle frontal gyrus, left angular gyrus, precuneus, superior lateral occipital cortex, and posterior cingulate cortex.

The dPI appeared to be more functionally connected with the left frontal pole, left superior frontal gyrus, left middle frontal gyrus, middle temporal areas, postcentral gyrus, left angular gyrus, precuneus, and occipital regions. The vPI had stronger functional connections to the right pars opercularis, SMA, right orbitofrontal cortex, peri-insular operculum, temporal pole, anterior superior temporal gyrus, auditory and peri-auditory areas, SMG, anterior cingulate cortex, right putamen, right globus pallidum, and amygdala. Overall, it appears that the dorsal–ventral comparative connectivity pattern of the posterior insula was the inverse pattern of the anterior and middle subregions.

### 3.5. Interhemispheric

**Figure 12:**
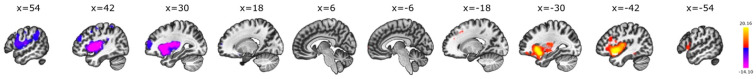
Left vs. right functional connectivity of insular subregions. Hot colors indicate areas more positively activated for the left hemisphere, while cold colors indicate areas more positively activated for the right hemisphere.

When comparing the interhemispheric differences (Figure 12), stronger functional connectivity was observed mainly between the ipsilateral regions. Overall, the left insula had stronger functional connections with the ipsilateral frontal pole, superior frontal gyrus, middle frontal gyrus, pars triangularis, pars opercularis, orbitofrontal cortex, frontal operculum, and paracingulate cortex. The right insula had stronger connections with the ipsilateral pars opercularis, precentral gyrus, peri-insular opercula, posterior SMG, angular gyrus, and putamen.

More specifically, the left dAI correlated strongly with the ipsilateral frontal pole, superior frontal gyrus, middle frontal gyrus, pars triangularis, pars opercularis, orbitofrontal cortex, frontal operculum, posterior middle temporal gyrus, angular gyrus, superior lateral occipital cortex, and paracingulate gyrus. On the other hand, the right dAI had stronger functional connections with the ipsilateral precentral gyrus, peri-insular opercula, SMG, putamen, amygdala, and bilateral planum temporale.

The left dMI was more functionally correlated with the ipsilateral frontal operculum while the right dMI connected more strongly with the ipsilateral postcentral gyrus.

Both the right and left dPI were positively correlated to their respective ipsilateral Heschl’s gyrus.

The right vAI correlated with the ipsilateral frontal pole, middle frontal gyrus, pars opercularis, orbitofrontal cortex, and angular gyrus.

In summary, we identified distinct functional connectivity patterns among the insular subregions. All the insular subregions displayed connectivity with the SMA, frontal, temporal, and parietal opercula, right temporal pole, anterior superior temporal gyrus, planum temporale, planum polare, Heschl’s gyrus, anterior cingulate cortex, left putamen, and amygdala, indicating a shared involvement in multimodal processing and in integrating diverse types of sensory information and coordinating responses related to motor control and emotional regulation. The dAI showed strong correlations with the frontal, superior temporal, and parietal regions, ACC, SMA, and SMG. The hemispheric differences revealed that the left dAI primarily correlated with the ventral frontal areas, while the right dAI was preferentially connected to the ipsilateral orbitofrontal cortex. The dMI exhibited similar correlations but included more parietal regions and lacked connections to the nucleus accumbens. The dPI shared connections with the dAI and additional regions such as the parahippocampal gyrus and hippocampus, with unique connections to the contralateral temporal occipital fusiform cortex and the supracalcarine cortex. The vAI was linked with the paracingulate gyrus, ACC, orbitofrontal cortex, and frontal opercula, also demonstrating hemispheric-specific connections. The vMI was primarily correlated with the ACC, while the vPI was connected to the ACC, superior temporal gyrus, SMG, postcentral gyrus, and SMA. The intra-insular connectivity analyses revealed that the anterior subregions were more functionally connected to the frontal regions, while the posterior subregions were connected more with the temporal and parietal areas. The dorsal insula regions showed more connections to the inferior frontal areas and pre- and postcentral gyri, auditory regions, SMG, ACC, and putamen, whereas the ventral regions were linked to the medial frontal and temporal gyri, Posterior Cingulate Gyrus (PCC), precuneus, and occipital regions. The interhemispheric comparisons indicated a stronger functional connectivity within the ipsilateral regions for both hemispheres.

## 4. Discussion

### 4.1. Cortical Functional Connectivity

The present study investigates the resting-state functional connectivity of an anatomically parcellated insula. Previous studies have suggested a rostro-caudal organization connectivity pattern where the anterior insula is functionally connected to the frontal and anterior cingulate areas while the posterior insula is connected with the sensorimotor regions [15]. Some studies have also subdivided the anterior insula into two additional regions: the ventral anterior subregion, which correlates with the limbic areas, and the dorsal anterior subregion, which is mostly connected to the frontal areas [16]. Our findings reproduce, in large part, these previous results while reporting novel insights as well. Mainly, we show novel correlations with the limbic and subcortical regions such as the nucleus accumbens, caudate nucleus, putamen, globus pallidus, and thalamus, as well as more detailed coactivations between the insular subregions and the anterior and posterior cingulate gyri.

The dAI showed connectivity with the peri-insular opercula and the frontal, superior temporal, and lateral parietal cortical areas. It is also essentially the only region that was not anticorrelated with the middle–superior frontal areas, which solidifies its inclination to the frontal areas. The dAI is known to be a central node in the salience network as showcased by its strong connection with the ACC [25]. Indeed, the dAI displayed the strongest correlation value with the ACC; the dAI’s involvement with the salience network will be discussed in a subsequent section. It has been hypothesized that the dAI is functionally connected to a cognitive control network which includes the anterior cingulate cortex, medial superior frontal cortex, anterior insula, frontal operculum, and anterior prefrontal cortex [56]. Our results are concordant with these findings, which demonstrated sustained activity in the dAI and ACC during goal-directed behavior in cognitive tasks [25,56,57,58]. This network potentially interacts with the salience network through the initiation of salient stimuli that are useful in completing goal-directed cognitive tasks. Therefore, the dAI may act as a transitional node that switches the current cognitive state from a salient stimuli-aware state to a higher-level functional cognitive central executive network state. Hence, somatosensory and emotional stimuli can form the basis of subsequent goal-oriented behavior [16]. These results are also concordant with those from structural connectivity studies [5,6,7,8,9,10]. The dAI’s connectivity with frontal areas also conveys its role in language processing. More specifically, a meta-analysis revealed that the left anterior insula was consistently activated during speech production tasks. Indeed, it has been proposed that the anterior insula mediates the motor aspects of language when considering lesional, stroke, and electrical stimulation studies. Functional imaging studies also support these findings [59]. Furthermore, our results show that both the dMI and vMI have significant frontal connections; it has been reported that the vMI is also involved in expressive language tasks [60]. As stated in the results section, these two insular regions have extended functional connections to frontal lobes, including to the pars triangularis and pars opercularis, which form Broca’s area. Hence, these results support the left insula’s involvement in language expression/production and articulation [60,61].

The dMI’s main connectivity profile encompasses the peri-auditory areas, temporal and parietal opercula, SMA, M1, and ACC. It also has broader parietal connections when compared with its ventral counterpart. These results are concordant with our previous work on structural connectivity [9]. The dMI’s connectivity profile shares more similarities with the anterior and posterior subdivisions than the vMI. This suggests that the dMI might serve as the main transitional area. However, the vMI is a smaller region formed of two subregions (L15 and R15). In other studies, the area occupied by the dMI may have been included in their appellation of the posterior insula [14,15,16,62]. When we compare its resting-state functional connectivity profile, it is concordant with these studies, but the dMI is the region with broader connections to secondary somatosensory cortices while the dPI is more connected to the SMA, primary motor cortex, and primary somatosensory cortex. The dorsal insula is known to be involved in pain processing as it is activated during noxious stimuli [63,64]. Additionally, thermoalgesic stimuli were found to activate the middle–posterior insula. This suggests that the dPI oversees basic painful stimuli processing while the dMI regulates its subjective discriminative property [65]. Pain stimuli finish their course in the vAI, where emotional features are added to the interpretation, rendering the experience more complete [66,67]. Due to the anatomical proximity of the dAI, a painful stimulus is said to be more intense due to the involvement of the salience network. Indeed, the perception of pain is increased when our attention is focused on it [64,66] and is decreased when a distraction is present [68].

Apart from connections to the parietal areas, the dPI is highly connected to the peri-auditory areas on the superior temporal gyrus, which includes Heschl’s gyrus, the planum temporale, and planum polare. These results are consistent with our previous work on the insula’s structural connectivity [9]. Together with the dMI, the dorsal middle–posterior insula appears to mediate auditory stimuli. Indeed, an fMRI meta-analysis revealed that the left dorsal middle insula was preferentially activated during speech perception tasks [60]. This can be explained by the subregion’s extensive connections to auditory areas. Our parcellation reveals that the main insular subregion connected to the peri-auditory areas is the dPI, closely followed by the vPI and dMI. The left hemisphere was also found to be preferentially activated in those respective regions. This is concordant with electrophysiologic work performed on epileptic patients [69]. The posterior insula was found to respond to auditory stimuli, similar to what is observed in Heschl’s gyrus. The posterior insula responded to fundamental frequencies, a response that was not observed in the anterior insula. It also reacted more to non-emotional sounds whereas the anterior insula responded strongly to emotional sound content. The same results were observed upon electrical stimulation of the posterior insula [70]. These results are also supported by lesion cases where auditory agnosia occurred upon posterior insular damage [71,72]. The right insula’s preference for parietal areas could be explained by the presence of the lateralized attention network in right-handed subjects. Indeed, lesions in the posterior insula have been reported to cause hemispatial neglect in patients [73].

The vAI is widely connected to limbic regions, which is concordant with previous studies suggesting its involvement in emotional stimuli processing [19,63]. Indeed, these findings mirror those of the previously mentioned study on sound processing in which the anterior insula responded strongly to emotional sound stimuli [70]. The vAI is also connected to the auditory cortical areas but to a lesser extent than the posterior insula. This transitional gradient of connectivity suggests that sounds are first processed in the posterior insula and gradually cross the insula in a posterior–anterior fashion to have more complex information such as emotional content extracted. The insula is therefore proposed to serve as an integrative hub to combine external and internal stimuli to create a complete interpretation of the stimulus. This pattern of connectivity can also be applied to pain processing, as discussed previously. Our findings show that the ventral-most region of the vAI is highly connected to the limbic regions, making it the main hub for emotional and affective processing such as risky decision-making, fairness evaluation, and empathy, which often involve the amygdala and prefrontal cortex [74,75,76,77].

In the vPI, contrary to our structural study [9], no connections were found with the occipital cortex. We hypothesize that this could be due to the patients having their eyes closed during MRI acquisition and therefore reduced activation in their occipital cortex.

### 4.2. Limbic and Subcortical Connectivity

Thalamus. Contrary to the structural findings, our results as well as those of previous rsfMRI studies reveal scarce to no connections to the thalamus [8,78]. One of its main roles is as a relay hub for sensory stimuli. Therefore, the connections we observed predominantly in the dAI could be explained by the thalamus’ involvement in processing salient information [79,80,81].

Putamen. Our findings regarding the connectivity of the putamen mirror those reported in our previous structural work [8]. Due to its anatomical proximity, it is no surprise that it is extensively connected to the insula. The proposed functions linking the two structures derive from functional imaging studies and include speech production, pain processing, drug addiction, and non-motor Parkinson’s disease symptoms [82,83,84,85,86,87].

Hippocampus. Our previous work demonstrated the structural connectivity between the hippocampus and the insula [8]; moreover, several other structural and functional studies investigating different pathologies have also linked these two regions. Further support for insular and hippocampal connections was shown in stereo-electroencephalography recordings, where the insula was generally the first area of the propagation of hippocampal seizures [88,89]. A post-traumatic stress disorder MRI review revealed the decreased structural brain volume of both these regions, while showing the decreased functional activity of the hippocampus and increased functional activity of the insula [90]. Both these regions also appear to be affected in schizophrenia, as a study showed widespread dysconnectivity between the posterior insula and hippocampus [91]. Similarly, reduced and disrupted functional connectivity was reported between the olfactory network, including the insula, and the hippocampus in patients with Alzheimer’s disease [92].

Globus pallidum. The globus pallidum has been shown to have functional connections with the left anterior insula and plays a role in speech production and articulation, which mirrors our results [93,94]. It has therefore been proposed that a pallido-insular functional connection plays a role in the fluidity of speech [95].

Caudate nucleus. The caudate nucleus was shown to be functionally connected to the anterior insula in our study, which mirrors the dense connectivity observed in previous structural and functional imaging studies [8,96]. The caudate nucleus is known to have an important role in cognitive and emotional processes [97,98]. Interestingly, these processes are also highly integrated with the anterior insula [19]. Functional imaging studies revealed that the dorsal anterior insula is prominently involved in cognition, attention, and decision-making while the ventral anterior insula seems to play a role in emotional processes [19]. The anterior insula and the caudate nucleus also seem to have integrative roles in processing the affective component of pain [99,100,101], which is supported by the substantial functional connections observed between both regions during painful tasks [96,99,101].

Amygdala. The amygdala has been largely linked to emotional integration, including reward processing and motivation, as well as memory functions [102]. In this regard, fMRI studies have revealed connections between the insula and the amygdala in resting-state paradigms and during the experience of anxiety [103,104]. Both areas were shown to be activated by emotional stimuli and risky decision-making and seem to be implicated in the integration of interoception and social cognition [105,106,107]. Moreover, our group previously reported structural connections between the two regions [8]. It is therefore not surprising that, in the current study, we detected rich functional connections between all six subregions of the insula and the amygdala.

Nucleus accumbens. The nucleus accumbens plays a primordial role in motivational and emotional processes. Functional connectivity studies have revealed the activation of the nucleus accumbens during impulsive and risk-taking behaviors and reward tasks [108]. Interestingly, all these functions have also been previously linked to the insula [108,109,110,111]. In the current study, we observed a functional correlation between many insular subregions and the nucleus accumbens, results once again supported by those of our previous structural work [8]. The connections between both regions could constitute an integrated circuit for their overlapping roles.

Interhemispheric differences. The involvement of the insula in specific functions, such as language and addiction, may explain the observed interhemispheric differences, as it involves different networks. Indeed, the left dAI showed the strongest positive correlations with frontal regions, concurring with previous functional and structural studies [7,15,21] as well as anatomical observations and hypotheses previously described regarding interoception, emotional processing, cognitive control, and addiction [112,113,114,115].

### 4.3. Comparison with Structural Connectivity

Since we used the same ROIs as in our previous structural work [8,9], it is possible to compare the results with the current resting-state functional connectivity. We observe a similar connectivity pattern in both modalities, where the dAI areas are mainly connected to frontal regions and temporal regions, such as the inferior frontal gyrus (pars triangularis and pars opercularis), precentral gyrus, planum temporale, planum polare, and Heschl’s gyrus. The dAI also has similar connections with the limbic and subcortical regions in both modalities including the ACC, putamen, and globus pallidum. The dMI has connections in both modalities with the superior and middle frontal gyri, precentral gyrus, temporal areas, ACC, PCC, putamen, globus pallidus, nuclei accumbens, and amygdala. The dPI was found to have structural and functional connections with the middle frontal gyrus, precentral and postcentral gyri, temporal areas, superior lateral occipital cortex, posterior parahippocampal gyrus, ACC, putamen, globus pallidus, and hippocampus. The vAI has similar connections in both modalities with the frontal orbital cortex, temporal areas such as the temporal pole, ACC, caudate nuclei, putamen, and globus pallidus. The vMI shares similar connections in structural and functional modalities with the frontal orbital cortex, anterior superior temporal gyrus, planum polare, anterior middle temporal gyrus, temporal pole, ACC, thalamus, caudate nuclei, putamen, and globus pallidus. Finally, the vPI shows similar structural and functional connectivity with the superior and middle frontal gyri, the precentral and postcentral gyri, angular gyrus, precuneus, superior temporal gyrus, planum temporale, planum polare, Heschl’s gyrus, temporal pole, superior lateral occipital cortex, ACC, caudate nuclei, putamen, hippocampus, amygdala, and nuclei accumbens. These results are consistent with each other (structural vs. rsFC) and the literature [63] regarding the connectivity gradient of the insula where anterior regions are more connected to the frontal, temporal, limbic, and subcortical areas, while the posterior regions are more connected with the parietal and occipital areas as well as the limbic and subcortical areas. Interestingly, no specific connections were only found in one modality, which shows that our method and results are congruent. An interhemispheric comparison was not feasible since interhemispheric connectivity in our structural work was not possible at the time of its completion.

### 4.4. Subdivisions of the Insula

Using a multimodal approach combining cortical myelin, cortical thickness, resting-state, and functional task findings, Glasser et al. [22] identified 13 subregions in the insular-fronto-opercular region, 6 of which were part of the insula per se (parainsular cortex, insula granular, posterior insular areas Pol1 and Pol2, middle insular area, anterior ventral insula, and anterior agranular insular complex). While it might have been interesting to study the distinct functional connectivity of their insular areas using their parcellation, we instead used the insular subdivisions (19 ROIs) from our previous structural connectivity work [8,9]. Interestingly, based on the connectivity patterns we observed for each of the 19 insular subdivisions, we were also able to identify 6 main subregions with distinct functional profiles. These regions overlapped to some extent with the six regions identified by Glasser et al. [22]. A brief comparison of the corresponding subregions is shown in Table 2. More detailed comparisons are difficult since the main focus of Glasser et al. [22] was the parcellation of the cortex (including the insula) rather than extracting a connectivity profile.

Furthermore, Tian and Zalesky [116] investigated which of the bipartite or tripartite parcellations provided the most parsimonious model of insula functional connectivity diversity and were unable to provide a conclusion. They instead found that such a model was strongly influenced by the variability between participants and the extent to which the functional data are spatially smoothed. They also report twelve studies that examined the functional connectivity profile and parcellation of the insula (see Tian and Zalesky [116], Table 1). These studies divided the insula into 2 to 12 clusters, while excluding whole-brain parcellation studies (e.g., Glasser et al. [22]). Finally, they conclude that the functional connectivity diversity of the insula could be characterized parsimoniously as a continuum, rather than persisting to try to define the optimal number of insular subregions. For this reason, we believe that our work contributes to the wealth of knowledge and diversity of insular divisions and their connectivity.

### 4.5. Functional Significance of Insular Subregions in Relation to Brain Function and Disease

An understanding the functional connectivity of insular subdivisions can help us better understand pathologies that selectively affect certain subregions of the insula such as isolated insular strokes or focal insular epilepsies and surgeries. Our group has notably contributed over the last two decades to improving our understanding of insular epilepsies. Using stereoencephalography sampling various subregions of the insula, we have shown that insular seizures can exhibit distinct semiological symptoms and signs depending on the subinsular region involved at seizure onset [117,118], largely explained by the functional subdivision of the insula and the propagation of the seizure discharge preferential to extra-insular areas that are structurally connected to that particular insular subregion [8,9]. We have also shown that ischemic damage or surgical removal of part of the insula (to remove a tumor or for seizure control) is frequently associated with different neurological/neuropsychological complications (thankfully most often transient or mild in intensity) including loss of hunger [119], decreased appetite [120], unpleasant odor processing [121], hyperacusis [122], thermal nociceptive deficits [123], altered risky decision-making [124], interference in affective information [125], and social information processing [126]. A better description of the functional connectivity of distinct insular subregions can thus help us better understand certain clinical observations. One could also surmise that such information could be practical to guide the selective target selection of novel therapeutic interventions such as deep brain stimulation or transcranial magnetic stimulation for various conditions involving insular dysfunction such as addiction, thermal pain, anorexia, etc. [123,127,128].

### 4.6. Limitations

Functional connectivity is sensitive to confounding factors, mainly head motions and physiological artifacts, such as respiratory and cardiac rhythms. In addition, activation patterns may be state-dependent on the participant’s acquisition scan, where estimates can be influenced by whether eyes are open or closed, passive fixation, stress, and slow or long respirations. These can have a direct inference on the interpretation of observed activation differences. Although there are several tools to control these effects, none are perfect, and each has strengths and weaknesses, hence the importance of balancing between a decrease in the effect of noise and a temporal loss of a degree of freedom. Here, we have tried to reach such a balance to the best of our abilities, although we cannot exclude the influence of such artifacts in our data. These artifacts, if not managed, can mimic the interpreted effect. Thus, rsFC is difficult to interpret on its own because it is an indirect relative measure of neural activity fluctuation. Therefore, it is useful as an exploratory and hypothetical approach and could be coupled with integrative methods for further investigation [129]. Finally, we discussed the limitations of our insular subdivisions in our previous work [9]; their inner transitional formation and relay roles could translate some temporal correlation signals that extend more towards surrounding areas.

## 5. Conclusions

In conclusion, following our tractography work, refined parcellation of the insula in the current rsfMRI study provides an intricate functional connectivity profile. Our findings are consistent with the gross differential connectivity patterns found in prior functional studies, using a traditional bipartite and tripartite division of the insula. We also provide a more detailed picture with specific differences in the dorsal and ventral areas of the anterior, middle, and posterior subregions as well as interhemispheric differences. We reveal novel connections with the limbic and subcortical regions, highlighting the insula’s involvement in complex functional networks, and its crucial role as a hub in mediating transitions between cognitive and emotional states. This detailed characterization of the insula’s functional connectivity matrix could help us understand the functional networks behind altered functions in pathological conditions involving the insula.

## Figures and Tables

**Table 1:** Insular subregion ROI composition based on their functional connectivity patterns.

Insular Subregion	ROI
Dorsal anterior	6, 7, 11, 12, 13
Ventral anterior	16, 17, 19
Dorsal middle	2, 5, 10
Ventral middle	15
Dorsal posterior	1, 3, 4
Ventral posterior	8, 9, 14, 18

**Table 2:** Brief comparison of the multimodal insular subdivision (Glasser et al. 2016 [22]) and our current insular subdivision.

Multimodal Insular Subdivision (Glasser et al. 2016 [22])	Current Insular Subdivision
AVI (anterior ventral insular area)	dAI
AAIC (anterior agranular insular complex)	vAI
Middle insular area (MI)	dMI
Insula granular (Ig)	dPI
Posterior insular areas (Pol1 and Pol2)	Part of vMI and vPI
Parainsular cortex (PI)	vPI

## Data Availability

The data that support the findings of this study are available on reasonable request from the corresponding author, D.K.N. The data are not publicly available due to privacy and ethical restrictions.

## Supplementary Materials

The following supporting information can be downloaded at https://www.mdpi.com/article/10.3390/brainsci14080742/s1, Figure S1: Left connectivity profile of 19 insular subregions; Figure S2: Right connectivity profile of 19 insular subregions; Table S1: Positive (+; green) and negative (−; red/pink) connectivity correlations between subregions of major lobes and insular subregions. r, right; l, left; FP, frontal pole; SFG, superior frontal gyrus; MidFG, middle frontal gyrus; IFG, inferior frontal gyrus; tri, triangularis; oper, opercularis; PreCG, precentral gyrus; MedFC, median frontal cortex; SMA, supplementary motor area; FOrb, orbitofrontal cortex; FO, frontal opercula. TP, temporal pole; aSTG, anterior superior temporal gyrus; pSTG, posterior superior temporal gyrus; aMTG, anterior middle temporal gyrus; pMTG, posterior middle temporal gyrus; toMTG, temporooccipital middle temporal gyrus; aITG, anterior inferior temporal gyrus; pITG, posterior inferior temporal gyrus; toITG, temporooccipital inferior temporal gyrus; aTFusC, anterior temporal fusiform cortex; pTFusC, posterior temporal fusiform cortex; TOfusC. Temporal occipital fusiform cortex; CO, central opercula; PP, planum polare; HG, Heschel’s gyrus; PT, planum temporale; PostCG, postcentral gyrus; SPL, superior parietal lobule; aSMG, anterior supramarginal gyrus; pSMG, posterior supramarginal gyrus; AG, angular gyrus; PO, parietal opercula; sLOC, superior lateral occipital cortex; iLOC, inferior lateral occipital cortex; OFusG, occipital fusiform gyrus; SCC, subcallosal cingulate cortex; OP, occipital pole; PaCiG, paracingulate gyrus; AC, anterior cingulate cortex; PC, posterior cingulate cortex; aPaHC, anterior parahippocampal cortex; pPaHC, posterior parahippocampal cortex. Table S2. Peak insular subregion cluster coordinates (*p* < 0.001 FDR corrected at cluster level) for all 6 ROIs in both hemispheres. a. Left dorsal anterior insula; b. right dorsal anterior insula; c. left dorsal middle anterior insula; d. right dorsal middle anterior insula; e. left dorsal posterior insula; f. right dorsal posterior insula; g. left ventral anterior insula; h. right ventral anterior insula; i. left ventral middle insula; j. right ventral middle insula; k. left ventral posterior insula; l. right ventral posterior insula; m. left vs. right insula n. left dorsal anterior vs. left dorsal posterior; o. right dorsal anterior vs. right dorsal posterior; *p*. left ventral anterior vs. left ventral posterior; q. right ventral anterior vs. right ventral posterior; r. left dorsal vs. left ventral insula; s. right dorsal vs. right ventral insula. Figure S3. ROI-to-ROI connections of the left dAI presented as a connectome ring. Figure S4. ROI-to-ROI connections of the right dAI presented as a connectome ring. Figure S5. ROI-to-ROI connections of the left dMI presented as a connectome ring. Figure S6. ROI-to-ROI connections of the right dMI presented as a connectome ring. Figure S7. ROI-to-ROI connections of the left dPI presented as a connectome ring. Figure S8. ROI-to-ROI connections of the right dPI presented as a connectome ring. Figure S9. ROI-to-ROI connections of the left vAI presented as a connectome ring. Figure S10. ROI-to-ROI connections of the right vAI presented as a connectome ring. Figure S11. ROI-to-ROI connections of the left vMI presented as a connectome ring. Figure S12. ROI-to-ROI connections of the right vMI presented as a connectome ring.

## References

[1] Stephani C., Fernandez-Baca Vaca G., Maciunas R., Koubeissi M., Luders H.O., Lüders H.O. (2011). Functional Neuroanatomy of the Insular Lobe. Brain Struct. Funct..

[2] Ture U., Yasargil D.C., Al-Mefty O., Yasargil M.G., Türe U., Yaşargil D.C., Al-Mefty O., Yaşargil M.G. (1999). Topographic Anatomy of the Insular Region. J. Neurosurg..

[3] Flynn F.G. (1999). Anatomy of the Insula Functional and Clinical Correlates. Aphasiology.

[4] Augustine J.R. (1985). The Insular Lobe in Primates Including Humans. Neurol. Res..

[5] Cloutman L.L., Binney R.J., Drakesmith M., Parker G.J.M., Lambon Ralph M.A. (2012). The Variation of Function across the Human Insula Mirrors Its Patterns of Structural Connectivity: Evidence from in Vivo Probabilistic Tractography. Neuroimage.

[6] Cerliani L., Thomas R.M., Jbabdi S., Siero J.C.W., Nanetti L., Crippa A., Gazzola V., D’Arceuil H., Keysers C. (2012). Probabilistic Tractography Recovers a Rostrocaudal Trajectory of Connectivity Variability in the Human Insular Cortex. Hum. Brain Mapp..

[7] Jakab A., Molnár P.P., Bogner P., Béres M., Berényi E.L., Molnar P.P., Bogner P., Beres M., Berenyi E.L. (2012). Connectivity-Based Parcellation Reveals Interhemispheric Differences in the Insula. Brain Topogr..

[8] Ghaziri J., Tucholka A., Girard G., Boucher O., Houde J.C., Descoteaux M., Obaid S., Gilbert G., Rouleau I., Nguyen D.K. (2018). Subcortical Structural Connectivity of Insular Subregions. Sci. Rep..

[9] Ghaziri J., Tucholka A., Girard G., Houde J.C., Boucher O., Gilbert G., Descoteaux M., Lippé S., Rainville P., Nguyen D.K. (2017). The Corticocortical Structural Connectivity of the Human Insula. Cereb. Cortex.

[10] Nomi J.S.S., Schettini E., Broce I., Dick A.S.S., Uddin L.Q.Q. (2017). Structural Connections of Functionally Defined Human Insular Subdivisions. Cereb. Cortex.

[11] Deco G., Jirsa V.K., McIntosh A.R. (2011). Emerging Concepts for the Dynamical Organization of Resting-State Activity in the Brain. Nat. Rev. Neurosci..

[12] Honey C.J., Sporns O., Cammoun L., Gigandet X., Thiran J.P., Meuli R., Hagmann P. (2009). Predicting Human Resting-State Functional Connectivity from Structural Connectivity. Proc. Natl. Acad. Sci. USA.

[13] van den Heuvel M.P., Mandl R.C.W.W., Kahn R.S., Hulshoff Pol H.E., Mandl C.W., Kahn S., Heuvel M.P., van den Pol H.E.H., van den Heuvel M.P., Mandl R.C.W.W. (2009). Functionally Linked Resting-State Networks Reflect the Underlying Structural Connectivity Architecture of the Human Brain. Hum. Brain Mapp..

[14] Taylor K.S., Seminowicz D.A., Davis K.D. (2009). Two Systems of Resting State Connectivity between the Insula and Cingulate Cortex. Hum. Brain Mapp..

[15] Cauda F., Agata F.D., Sacco K., Duca S., Geminiani G., Vercelli A., D’Agata F., Sacco K., Duca S., Geminiani G. (2011). Functional Connectivity of the Insula in the Resting Brain. Neuroimage.

[16] Deen B., Pitskel N.B., Pelphrey K.A. (2011). Three Systems of Insular Functional Connectivity Identified with Cluster Analysis. Cereb. Cortex.

[17] Kelly C., Toro R., Di Martino A., Cox C.L., Bellec P., Castellanos F.X., Milham M.P. (2012). A Convergent Functional Architecture of the Insula Emerges across Imaging Modalities. Neuroimage.

[18] Chang L.J., Yarkoni T., Khaw M.W., Sanfey A.G. (2013). Decoding the Role of the Insula in Human Cognition: Functional Parcellation and Large-Scale Reverse Inference. Cereb. Cortex.

[19] Kurth F., Zilles K., Fox P.T., Laird A.R., Eickhoff S.B. (2010). A Link between the Systems: Functional Differentiation and Integration within the Human Insula Revealed by Meta-Analysis. Brain Struct. Funct..

[20] Uddin L.Q., Kinnison J., Pessoa L., Anderson M.L. (2014). Beyond the Tripartite Cognition–Emotion–Interoception Model of the Human Insular Cortex. J. Cogn. Neurosci..

[21] Cauda F., Costa T., Torta D.M.E., Sacco K., D’Agata F., Duca S., Geminiani G., Fox P.T., Vercelli A. (2012). Meta-Analytic Clustering of the Insular Cortex: Characterizing the Meta-Analytic Connectivity of the Insula When Involved in Active Tasks. Neuroimage.

[22] Glasser M.F., Coalson T.S., Robinson E.C., Hacker C.D., Harwell J., Yacoub E., Ugurbil K., Andersson J., Beckmann C.F., Jenkinson M. (2016). A Multi-Modal Parcellation of Human Cerebral Cortex. Nature.

[23] Morel A., Gallay M.N., Baechler A., Wyss M., Gallay D.S. (2013). The Human Insula: Architectonic Organization and Postmortem MRI Registration. Neuroscience.

[24] Vercelli U.G.O., Diano M., Costa T., Nani A., Duca S., Geminiani G., Vercelli A., Cauda F. (2015). Node Detection Using High-Dimensional Fuzzy Parcellation Applied to the Insular Cortex. Neural Plast..

[25] Menon V., Uddin L.Q. (2010). Saliency, Switching, Attention and Control: A Network Model of Insula Function. Brain Struct. Funct..

[26] Paulus M.P., Stein M.B. (2006). An Insular View of Anxiety. Biol. Psychiatry.

[27] Whitfield-Gabrieli S., Nieto-Castanon A. (2012). Conn: A Functional Connectivity Toolbox for Correlated and Anticorrelated Brain Networks. Brain Connect.

[28] Calhoun V.D., Wager T.D., Krishnan A., Rosch K.S., Seymour K.E., Nebel M.B., Mostofsky S.H., Nyalakanai P., Kiehl K. (2017). The Impact of T1 versus EPI Spatial Normalization Templates for FMRI Data Analyses. Hum. Brain Mapp..

[29] Ashburner J., Friston K.J. (2005). Unified Segmentation. Neuroimage.

[30] Ashburner J. (2007). A Fast Diffeomorphic Image Registration Algorithm. Neuroimage.

[31] Andersson J.L.R., Hutton C., Ashburner J., Turner R., Friston K. (2001). Modeling Geometric Deformations in EPI Time Series. Neuroimage.

[32] Friston K.J., Ashburner J., Frith C.D., Poline J.-B., Heather J.D., Frackowiak R.S.J. (1995). Spatial Registration and Normalization of Images. Hum. Brain Mapp..

[33] Henson R., Buchel C., Josephs O., Friston K. (1999). The Slice-Timing Problem in Event-Related FMRI. Neuroimage.

[34] Sladky R., Friston K.J., Tröstl J., Cunnington R., Moser E., Windischberger C. (2011). Slice-Timing Effects and Their Correction in Functional MRI. Neuroimage.

[35] Power J.D., Mitra A., Laumann T.O., Snyder A.Z., Schlaggar B.L., Petersen S.E. (2014). Methods to Detect, Characterize, and Remove Motion Artifact in Resting State FMRI. Neuroimage.

[36] Demertzi A., Antonopoulos G., Heine L., Voss H.U., Crone J.S., de Los Angeles C., Bahri M.A., Di Perri C., Vanhaudenhuyse A., Charland-Verville V. (2015). Intrinsic Functional Connectivity Differentiates Minimally Conscious from Unresponsive Patients. Brain.

[37] Nieto-Castanon A. (2020). FMRI Denoising Pipeline. Handbook of Functional Connectivity Magnetic Resonance Imaging Methods in CONN.

[38] Behzadi Y., Restom K., Liau J., Liu T.T. (2007). A Component Based Noise Correction Method (CompCor) for BOLD and Perfusion Based FMRI. Neuroimage.

[39] Chai X.J., Castañón A.N., Öngür D., Whitfield-Gabrieli S. (2012). Anticorrelations in Resting State Networks without Global Signal Regression. Neuroimage.

[40] Friston K.J., Williams S., Howard R., Frackowiak R.S.J., Turner R. (1996). Movement-Related Effects in FMRI Time-Series. Magn. Reson. Med..

[41] Hallquist M.N., Hwang K., Luna B. (2013). The Nuisance of Nuisance Regression: Spectral Misspecification in a Common Approach to Resting-State FMRI Preprocessing Reintroduces Noise and Obscures Functional Connectivity. Neuroimage.

[42] Greicius M.D., Krasnow B., Reiss A.L., Menon V. (2003). Functional Connectivity in the Resting Brain: A Network Analysis of the Default Mode Hypothesis. Proc. Natl. Acad. Sci. USA.

[43] Fox M.D., Snyder A.Z., Vincent J.L., Corbetta M., Van Essen D.C., Raichle M.E. (2005). The Human Brain Is Intrinsically Organized into Dynamic, Anticorrelated Functional Networks. Proc. Natl. Acad. Sci. USA.

[44] Nieto-Castanon A. (2022). Preparing FMRI Data for Statistical Analysis. arXiv.

[45] Biswal B.B., Mennes M., Zuo X.-N., Gohel S., Kelly C., Smith S.M., Beckmann C.F., Adelstein J.S., Buckner R.L., Colcombe S. (2010). Toward Discovery Science of Human Brain Function. Proc. Natl. Acad. Sci. USA.

[46] Smith S.M., Fox P.T., Miller K.L., Glahn D.C., Fox P.M., Mackay C.E., Filippini N., Watkins K.E., Toro R., Laird A.R. (2009). Correspondence of the Brain’s Functional Architecture during Activation and Rest. Proc. Natl. Acad. Sci. USA.

[47] Nieto-Castanon A. (2020). Functional Connectivity Measures. Handbook of Functional Connectivity Magnetic Resonance Imaging Methods in CONN.

[48] Worsley K.J., Marrett S., Neelin P., Vandal A.C., Friston K.J., Evans A.C., Brain M. (1996). A Unified Statistical Approach for Determining Significant Signals in Images of Cerebral Activation. Hum. Brain Mapp..

[49] Nieto-Castanon A. (2020). Cluster-Level Inferences. Handbook of Functional Connectivity Magnetic Resonance Imaging Methods in CONN.

[50] Nieto-Castanon A. (2020). General Linear Model. Handbook of Functional Connectivity Magnetic Resonance Imaging Methods in CONN.

[51] Chumbley J., Worsley K., Flandin G., Friston K. (2010). Topological FDR for Neuroimaging. Neuroimage.

[52] Makris N., Goldstein J.M., Kennedy D., Hodge S.M., Caviness V.S., Faraone S.V., Tsuang M.T., Seidman L.J. (2006). Decreased Volume of Left and Total Anterior Insular Lobule in Schizophrenia. Schizophr. Res..

[53] Frazier J.A., Chiu S., Breeze J.L., Makris N., Lange N., Kennedy D.N., Herbert M.R., Bent E.K., Koneru V.K., Dieterich M.E. (2005). Structural Brain Magnetic Resonance Imaging of Limbic and Thalamic Volumes in Pediatric Bipolar Disorder. Am. J. Psychiatry.

[54] Desikan R.S., Ségonne F., Fischl B., Quinn B.T., Dickerson B.C., Blacker D., Buckner R.L., Dale A.M., Maguire R.P., Hyman B.T. (2006). An Automated Labeling System for Subdividing the Human Cerebral Cortex on MRI Scans into Gyral Based Regions of Interest. Neuroimage.

[55] Goldstein J.M., Seidman L.J., Makris N., Ahern T., O’Brien L.M., Caviness V.S., Kennedy D.N., Faraone S.V., Tsuang M.T. (2007). Hypothalamic Abnormalities in Schizophrenia: Sex Effects and Genetic Vulnerability. Biol. Psychiatry.

[56] Dosenbach N.U.F., Fair D.A., Miezin F.M., Cohen A.L., Wenger K.K., Dosenbach R.A.T., Fox M.D., Snyder A.Z., Vincent J.L., Raichle M.E. (2007). Distinct Brain Networks for Adaptive and Stable Task Control in Humans. Proc. Natl. Acad. Sci. USA.

[57] Lamichhane B., Dhamala M. (2015). The Salience Network and Its Functional Architecture in a Perceptual Decision: An Effective Connectivity Study. Brain Connect.

[58] Kelly A.M.C., Uddin L.Q., Biswal B.B., Castellanos F.X., Milham M.P. (2008). Competition between Functional Brain Networks Mediates Behavioral Variability. Neuroimage.

[59] Ackermann H., Riecker A. (2010). The Contribution(s) of the Insula to Speech Production: A Review of the Clinical and Functional Imaging Literature. Brain Struct. Funct..

[60] Oh A., Duerden E.G., Pang E.W. (2014). The Role of the Insula in Speech and Language Processing. Brain Lang..

[61] Boucher O., Rouleau I., Escudier F., Malenfant A., Denault C., Charbonneau S., Finet P., Lassonde M., Lepore F., Bouthillier A. (2015). Neuropsychological Performance before and after Partial or Complete Insulectomy in Patients with Epilepsy. Epilepsy Behav..

[62] van den Heuvel M.P., Hulshoff Pol H.E. (2010). Exploring the Brain Network: A Review on Resting-State FMRI Functional Connectivity. Eur. Neuropsychopharmacol..

[63] Uddin L.Q., Nomi J.S., Hébert-Seropian B., Ghaziri J., Boucher O. (2017). Structure and Function of the Human Insula. J. Clin. Neurophysiol..

[64] Tracey I., Mantyh P.W. (2007). The Cerebral Signature for Pain Perception and Its Modulation. Neuron.

[65] Singer T., Seymour B., O’Doherty J., Kaube H., Dolan R.J., Frith C.D. (2004). Empathy for Pain Involves the Affective but Not Sensory Components of Pain. Science.

[66] Lutz A., McFarlin D.R., Perlman D.M., Salomons T.V., Davidson R.J. (2013). Altered Anterior Insula Activation during Anticipation and Experience of Painful Stimuli in Expert Meditators. Neuroimage.

[67] Wiech K., Lin C.S., Brodersen K.H., Bingel U., Ploner M., Tracey I. (2010). Anterior Insula Integrates Information about Salience into Perceptual Decisions about Pain. J. Neurosci..

[68] Kucyi A., Davis K.D. (2015). The Dynamic Pain Connectome. Trends Neurosci..

[69] Mazzola L., Mauguière F., Isnard J. (2019). Functional Mapping of the Human Insula: Data from Electrical Stimulations. Rev. Neurol..

[70] Zhang Y., Zhou W., Wang S., Zhou Q., Wang H., Zhang B., Huang J., Hong B., Wang X. (2019). The Roles of Subdivisions of Human Insula in Emotion Perception and Auditory Processing. Cereb. Cortex.

[71] Bamiou D.-E.E., Musiek F.E., Luxon L.M. (2003). The Insula (Island of Reil) and Its Role in Auditory Processing. Literature Review. Brain Res. Brain Res. Rev..

[72] Habib M., Daquin G., Milandre L., Royere M.L., Rey M., Lanteri A., Salamon G., Khalil R. (1995). Mutism and Auditory Agnosia Due to Bilateral Insular Damage--Role of the Insula in Human Communication. Neuropsychologia.

[73] Golay L., Schnider A., Ptak R. (2008). Cortical and Subcortical Anatomy of Chronic Spatial Neglect Following Vascular Damage. Behav. Brain Funct..

[74] Singer T., Critchley H.D., Preuschoff K. (2009). A Common Role of Insula in Feelings, Empathy and Uncertainty. Trends Cogn. Sci..

[75] Cheng X., Zheng L., Li L., Zheng Y., Guo X., Yang G. (2017). Anterior Insula Signals Inequalities in a Modified Ultimatum Game. Neuroscience.

[76] Weller J.A., Levin I.P., Shiv B., Bechara A. (2009). The Effects of Insula Damage on Decision-Making for Risky Gains and Losses. Soc. Neurosci..

[77] Gasquoine P.G. (2014). Contributions of the Insula to Cognition and Emotion. Neuropsychol. Rev..

[78] Cauda F., Geminiani G., D’Agata F., Sacco K., Duca S., Bagshaw A.P., Cavanna A.E., Cavanna A.E. (2010). Functional Connectivity of the Posteromedial Cortex. PLoS ONE.

[79] Seeley W.W. (2019). The Salience Network: A Neural System for Perceiving and Responding to Homeostatic Demands. J. Neurosci..

[80] Uddin L.Q. (2014). Salience Processing and Insular Cortical Function and Dysfunction. Nat. Rev. Neurosci..

[81] Seeley W.W., Menon V., Schatzberg A.F., Keller J., Glover G.H., Kenna H., Reiss A.L., Greicius M.D. (2007). Dissociable Intrinsic Connectivity Networks for Salience Processing and Executive Control. J. Neurosci..

[82] Brooks J.C.W., Zambreanu L., Godinez A., Craig A.D., Tracey I. (2005). Somatotopic Organisation of the Human Insula to Painful Heat Studied with High Resolution Functional Imaging. Neuroimage.

[83] Price C.J. (2009). The Anatomy of Language: A Review of 100 FMRI Studies Published in 2009. Ann. N. Y. Acad. Sci..

[84] Akkermans S.E.A., Luijten M., van Rooij D., Franken I.H.A., Buitelaar J.K. (2018). Putamen Functional Connectivity during Inhibitory Control in Smokers and Non-Smokers. Addict. Biol..

[85] Naqvi N.H., Rudrauf D., Damasio H., Bechara A. (2007). Damage to the Insula Disrupts Addiction to Cigarette Smoking. Science.

[86] Christopher L., Koshimori Y., Lang A.E., Criaud M., Strafella A.P. (2014). Uncovering the Role of the Insula in Non-Motor Symptoms of Parkinson’s Disease. Brain.

[87] Criaud M., Christopher L., Boulinguez P., Ballanger B., Lang A.E., Cho S.S., Houle S., Strafella A.P. (2016). Contribution of Insula in Parkinson’s Disease: A Quantitative Meta-Analysis Study. Hum. Brain Mapp..

[88] Isnard J., Guénot M., Ostrowsky K., Sindou M., Mauguière F. (2000). The Role of the Insular Cortex in Temporal Lobe Epilepsy. Ann. Neurol..

[89] Isnard J., Guénot M., Sindou M., Mauguière F. (2004). Clinical Manifestations of Insular Lobe Seizures: A Stereo-Electroencephalographic Study. Epilepsia.

[90] Kunimatsu A., Yasaka K., Akai H., Kunimatsu N., Abe O. (2020). MRI Findings in Posttraumatic Stress Disorder. J. Magn. Reson. Imaging.

[91] Sheffield J.M., Rogers B.P., Blackford J.U., Heckers S., Woodward N.D. (2020). Insula Functional Connectivity in Schizophrenia. Schizophr. Res..

[92] Lu J., Testa N., Jordan R., Elyan R., Kanekar S., Wang J., Eslinger P., Yang Q.X., Zhang B., Karunanayaka P.R. (2019). Functional Connectivity between the Resting-State Olfactory Network and the Hippocampus in Alzheimer’s Disease. Brain Sci..

[93] Brown P., Williams D. (2005). Basal Ganglia Local Field Potential Activity: Character and Functional Significance in the Human. Clin. Neurophysiol..

[94] Wise R.J., Greene J., Büchel C., Scott S.K. (1999). Brain Regions Involved in Articulation. Lancet.

[95] Manes J.L., Parkinson A.L., Larson C.R., Greenlee J.D., Eickhoff S.B., Corcos D.M., Robin D.A. (2014). Connectivity of the Subthalamic Nucleus and Globus Pallidus Pars Interna to Regions within the Speech Network: A Meta-Analytic Connectivity Study. Hum. Brain Mapp..

[96] Robinson J.L., Laird A.R., Glahn D.C., Blangero J., Sanghera M.K., Pessoa L., Fox P.M., Uecker A., Friehs G., Young K.A. (2012). The Functional Connectivity of the Human Caudate: An Application of Meta-Analytic Connectivity Modeling with Behavioral Filtering. Neuroimage.

[97] di Martino A., Scheres A., Margulies D.S., Kelly A.M.C., Uddin L.Q., Shehzad Z., Biswal B., Walters J.R., Castellanos F.X., Milham M.P. (2008). Functional Connectivity of Human Striatum: A Resting State FMRI Study. Cereb. Cortex.

[98] Mueller S., Wang D., Pan R., Holt D.J., Liu H., RW K. (2015). Abnormalities in Hemispheric Specialization of Caudate Nucleus Connectivity in Schizophrenia. JAMA Psychiatry.

[99] Bushnell M.C., Ceko M., Low L.A. (2013). Cognitive and Emotional Control of Pain and Its Disruption in Chronic Pain. Nat. Rev. Neurosci..

[100] Borsook D., Upadhyay J., Chudler E.H., Becerra L. (2010). A Key Role of the Basal Ganglia in Pain and Analgesia—Insights Gained through Human Functional Imaging. Mol. Pain.

[101] Emmert K., Breimhorst M., Bauermann T., Birklein F., van de Ville D., Haller S. (2014). Comparison of Anterior Cingulate vs. Insular Cortex as Targets for Real-Time FMRI Regulation during Pain Stimulation. Front. Behav. Neurosci..

[102] Phillips R.G., LeDoux J.E. (1992). Differential Contribution of Amygdala and Hippocampus to Cued and Contextual Fear Conditioning. Behav. Neurosci..

[103] Roy A.K., Shehzad Z., Margulies D.S., Kelly A.M.C., Uddin L.Q., Gotimer K., Biswal B.B., Castellanos F.X., Milham M.P. (2009). Functional Connectivity of the Human Amygdala Using Resting State FMRI. Neuroimage.

[104] Baur V., Hänggi J., Langer N., Jäncke L. (2013). Resting-State Functional and Structural Connectivity within an Insula-Amygdala Route Specifically Index State and Trait Anxiety. Biol. Psychiatry.

[105] Viinikainen M., Jääskeläinen I.P., Alexandrov Y., Balk M.H., Autti T., Sams M. (2009). Nonlinear Relationship between Emotional Valence and Brain Activity: Evidence of Separate Negative and Positive Valence Dimensions. Hum. Brain Mapp..

[106] Smith B.W., Mitchell D.G.V., Hardin M.G., Jazbec S., Fridberg D., Blair R.J.R., Ernst M. (2009). Neural Substrates of Reward Magnitude, Probability, and Risk during a Wheel of Fortune Decision-Making Task. Neuroimage.

[107] Adolfi F., Couto B., Richter F., Decety J., Lopez J., Sigman M., Manes F., Ibáñez A. (2017). Convergence of Interoception, Emotion, and Social Cognition: A Twofold FMRI Meta-Analysis and Lesion Approach. Cortex.

[108] Salgado S., Kaplitt M.G. (2015). The Nucleus Accumbens: A Comprehensive Review. Ster. Funct. Neurosurg..

[109] Dambacher F., Sack A.T., Lobbestael J., Arntz A., Brugman S., Schuhmann T. (2013). Out of Control: Evidence for Anterior Insula Involvement in Motor Impulsivity and Reactive Aggression. Soc. Cogn. Affect Neurosci..

[110] Nachev P., Lopez-Sosa F., Gonzalez-Rosa J.J., Galarza A., Avecillas J., Pineda-Pardo J.A., Lopez-Ibor J.J., Reneses B., Barcia J.A., Strange B. (2015). Dynamic Risk Control by Human Nucleus Accumbens. Brain.

[111] Liu X., Hairston J., Schrier M., Fan J. (2011). Common and Distinct Networks Underlying Reward Valence and Processing Stages: A Meta-Analysis of Functional Neuroimaging Studies. Neurosci. Biobehav. Rev..

[112] Clark L., Studer B., Bruss J., Tranel D., Bechara A. (2014). Damage to Insula Abolishes Cognitive Distortions during Simulated Gambling. Proc. Natl. Acad. Sci. USA.

[113] Craig A.D. (2004). Human Feelings: Why Are Some More Aware than Others?. Trends Cogn. Sci..

[114] Craig A.D. (2003). Interoception: The Sense of the Physiological Condition of the Body. Curr. Opin. Neurobiol..

[115] Craig A.D. (2009). How Do You Feel--Now? The Anterior Insula and Human Awareness. Nat. Rev. Neurosci..

[116] Tian Y., Zalesky A. (2018). Characterizing the Functional Connectivity Diversity of the Insula Cortex: Subregions, Diversity Curves and Behavior. Neuroimage.

[117] Ryvlin P., Nguyen D.K. (2021). Insular Seizures and Epilepsies: Ictal Semiology and Minimal Invasive Surgery. Curr. Opin. Neurol..

[118] Levy A., Yen Tran T.P., Boucher O., Bouthillier A., Nguyen D.K. (2017). Operculo-Insular Epilepsy: Scalp and Intracranial Electroencephalographic Findings. J. Clin. Neurophysiol..

[119] Hébert-Seropian B., Boucher O., Jutras-Aswad D., Nguyen D.K. (2021). Uncommon Case of Complete Loss of Hunger Following an Isolated Left Insular Stroke. Neurocase.

[120] Hébert-Seropian B., Boucher O., Citherlet D., Roy-Côté F., Gravel V., Obaid S., Bouthillier A., Nguyen D.K. (2021). Decreased Self-Reported Appetite Following Insular Cortex Resection in Patients with Epilepsy. Appetite.

[121] Roy-Côté F., Zahal R., Frasnelli J., Nguyen D.K., Boucher O. (2021). Insula and Olfaction: A Literature Review and Case Report. Brain Sci..

[122] Boucher O., Turgeon C., Champoux S., Menard L., Rouleau I., Lassonde M., Lepore F., Nguyen D.K. (2015). Hyperacusis Following Unilateral Damage to the Insular Cortex: A Three-Case Report. Brain Res..

[123] Denis D.J., Marouf R., Rainville P., Bouthillier A., Nguyen D.K. (2016). Effects of Insular Stimulation on Thermal Nociception. Eur. J. Pain.

[124] Von Siebenthal Z., Boucher O., Rouleau I., Lassonde M., Lepore F., Nguyen D.K. (2017). Decision-Making Impairments Following Insular and Medial Temporal Lobe Resection for Drug-Resistant Epilepsy. Soc. Cogn. Affect. Neurosci..

[125] Citherlet D., Boucher O., Gravel V., Roy-Côté F., Bouthillier A., Nguyen D.K. (2020). The Effects of Insular and Mesiotemporal Lesions on Affective Information Processing: Preliminary Evidence from Patients with Epilepsy Surgery. Epilepsy Behav..

[126] Boucher O., Rouleau I., Lassonde M., Lepore F., Bouthillier A., Nguyen D.K. (2015). Social Information Processing Following Resection of the Insular Cortex. Neuropsychologia.

[127] Bergeron D., Obaid S., Fournier-Gosselin M.P., Bouthillier A., Nguyen D.K. (2021). Deep Brain Stimulation of the Posterior Insula in Chronic Pain: A Theoretical Framework. Brain Sci..

[128] Zangen A., Moshe H., Martinez D., Barnea-Ygael N., Vapnik T., Bystritsky A., Duffy W., Toder D., Casuto L., Grosz M.L. (2021). Repetitive Transcranial Magnetic Stimulation for Smoking Cessation: A Pivotal Multicenter Double-Blind Randomized Controlled Trial. World Psychiatry.

[129] Buckner R.L., Krienen F.M., Yeo B.T.T. (2013). Opportunities and Limitations of Intrinsic Functional Connectivity MRI. Nat. Neurosci..

