# Supplementary material for: Resting-State Functional Connectivity Profile of Insular Subregions
Source: Brain Sci. 2024 Jul 25;14(8):742. doi: 10.3390/brainsci14080742 (PMC11352390; doi:10.3390/brainsci14080742)
Supplement: Supplementary file 1 [file brainsci-14-00742-s001.zip › brainsci-3091005-supplementary.pdf]

Resting-state functional connectivity profile of insular subregions

Jimmy Ghaziri [1,2], Phillip Fei [3], Alan Tucholka [4,5], Sami Obaid [2], Olivier Boucher [2], Isabelle Rouleau [1,2], and Dang K. Nguyen [2,6,*]

[1] Département de psychologie, Université du Québec à Montréal, Montréal, Qc, Canada
[2] Centre de Recherche du Centre Hospitalier de l'Université de Montréal, Montréal, Qc, Canada
[3] Faculté de Médecine et des Sciences de la Santé, Université de Sherbrooke, Chicoutimi, Qc, Canada
[4] BarcelonaBeta Brain Research Center, Pasqual Maragall Foundation, Barcelona Spain
[5] Pixyl Medical, Grenoble, France
[6] Service de Neurologie, Centre Hospitalier de l'Université de Montréal (CHUM), Montréal, Qc, Canada

		dAI - Left	dAI - Right	vAI - Left	vAI - Right	dMI - Left	dMI - Right	vMI - Left	vMI - Right	dPI - Left	dPI - Right	vPI - Left	vPI - Right
	FP l					-	-			-	-	-	-
	FP r				+	-	-			-	-	-	-
	SFG l		-			-	-		-			-	-
	SFG r	+	+	+	+					-			
	MidFG l					-	-	-	-	-	-	-	-
	MidFG r					-	-	-	-	-	-	-	-
	IFG tri r	+	+		+				+				
	IFG oper l	+											
Frontal lobe	IFG oper r	+	+		+	+	+	+	+			+	+
	PreCG l	+	+			+	+	+	+	+	+	+	+
	PreCG r	+	+			+	+	+	+	+	+	+	+
	MedFC			+	+					+	+		
	SMA L	+	+	+	+	+	+	+	+	+	+	+	+
	SMA r	+	+	+	+	+	+	+	+	+	+	+	+
	FOrb l	+		+	+	+		+	+	+		+	
	FOrb r	+	+	+	+	+	+	+	+			+	+
	FO l	+	+	+	+	+	+	+	+	+	+	+	+
	FO r	+	+	+	+	+	+	+	+	+	+	+	+
	TP l	+		+		+	+	+	+	+	+	+	+
	TP r	+	+	+	+	+	+	+	+	+	+	+	+
	aSTG l	+	+	+	+	+	+	+	+	+	+	+	+
	aSTG r	+	+	+	+	+	+	+	+	+	+	+	+
	pSTG l		+			+	+	+	+	+	+	+	+
	pSTG r	+	+			+	+	+	+	+	+	+	+
	aMTG l	-	-			-		-				-	
Temporal lobe	aMTG r	-	-			-	-	-	-			-	-
	pMTG l	-	-			-	-		-			-	-
	pMTG r	-	-		+	-							
	toMTG r								+				+
	aITG l	-	-			-						-	
	aITG r	-	-		+	-							
	pITG l		-	-	-			-	-		-	-	-
	pITG r	-	-	-	-	-		-	-			-	-

	toITG l			-	-						-		
	toITG r			-	-								
	aTFusC l											+	
	pTFusC l			-	-								
	pTFusC r		-	-	-								
	TOFusC l				-								
	TOFusC r				-					+			
	CO l	+	+	+	+	+	+	+	+	+	+	+	+
	CO r	+	+	+	+	+	+	+	+	+	+	+	+
	PP l	+	+	+	+	+	+	+	+	+	+	+	+
	PP r	+	+	+	+	+	+	+	+	+	+	+	+
	HG l	+	+	+	+	+	+	+	+	+	+	+	+
	HG r	+	+	+	+	+	+	+	+	+	+	+	+
	PT l	+	+	+	+	+	+	+	+	+	+	+	+
	PT r	+	+	+	+	+	+	+	+	+	+	+	+
	PostCG l					+	+			+	+	+	+
	PostCG r				-	+	+			+	+	+	+
	SPL l				-	+	+						
	SPL r					+	+						+
	aSMG l	+	+			+	+	+	+	+		+	+
	aSMG r	+	+			+	+	+	+	+	+	+	+
Parietal lobe	pSMG l	+	+			+	+	+	+			+	+
	pSMG r	+	+		+	+	+	+	+			+	+
	AG l		-			-	-	-	-	-	-	-	-
	AG r				+	-				-	-		
	Precuneous	-	-			-	-	-	-				
	PO l	+	+	+	+	+	+	+	+	+	+	+	+
	PO r	+	+	+	+	+	+	+	+	+	+	+	+
	sLOC l	-	-	-	-	-	-	-	-	-	-	-	-
	sLOC r	-	-	-		-	-	-	-	-	-	-	-
	iLOC r	-	-		-								
	OFusG l				-								
	OFusG r	-		-	-								
Occipital lobe	SCC l										+		
	SCC r										+		
	OP r	-											
	PaCiG l	+		+	+			+					
	PaCiG r	+	+	+	+			+	+				
	atlas.AC	+	+	+	+	+	+	+	+	+	+	+	+
	atlas.PC			+	+	-	-						
	aPaHC l									+	+		
	aPaHC r						+			+	+		+
	pPaHC l		-							+	+		
	pPaHC r				+					+	+		+
	Thalamus r		+						+				
	Caudate l	+	+		+								
	Putamen l	+	+	+	+	+	+	+	+	+	+	+	+
	Putamen r	+	+	+	+	+	+	+	+		+	+	+
Subcortical and limbic areas	Pallidum l	+	+			+	+	+	+			+	+
	Pallidum r	+	+			+			+				
	Hippocampus l			+	+					+	+		

Hippocampus r	-			+					+	+		
Amygdala l	+	+	+	+	+	+	+	+	+	+	+	+
Amygdala r	+	+	+	+	+	+	+	+	+	+	+	+
Accumbens l	+	+	+	+	+			+			+	+
Accumbens r			+	+							+	

Table S1.: Positive (+; green) and negative (-; red/pink) connectivity correlations between subregions of major lobes and insular subregions. r, right; l, left FP, frontal pole**;** SFG, superior frontal gyrus; MidFG, middle frontal gyrus; IFG, inferior frontal gyrus; tri, triangularis; oper, opercularis; PreCG, precentral gyrus; MedFC, median frontal cortex; SMA, supplementary motor area; FOrb, orbitofrontal cortex; FO, frontal opercula. TP, temporal pole; aSTG, anterior superior temporal gyrus; pSTG, posterior superior temporal gyrus; aMTG, anterior middle temporal gyrus; pMTG, posterior middle temporal gyrus; toMTG, temporooccipital middle temporal gyrus; aITG, anterior inferior temporal gyrus; pITG, posterior inferior temporal gyrus; toITG, temporooccipital inferior temporal gyrus; aTFusC, anterior temporal fusiform cortex; pTFusC, posterior temporal fusiform cortex; TOfusC. Temporal occipital fusiform cortex; CO, central opercula; PP, planum polare; HG, Heschel's gyrus; PT, planum temporale; PostCG, postcentral gyrus; SPL, superior parietal lobule; aSMG, anterior supramarginal gyrus; pSMG, posterior supramarginal gyrus; AG, angular gyrus; PO, parietal opercula; sLOC, superior lateral occipital cortex; iLOC, inferior lateral occipital cortex; OFusG, occipital fusiform gyrus; SCC, subcallosal cingulate cortex; OP , occipital pole; PaCiG, paracingulate gyrus; AC, anterior cingulate cortex; PC, posterior cingulate cortex; aPaHC, anterior parahippocampal cortex; pPaHC, posterior parahippocampal cortex.

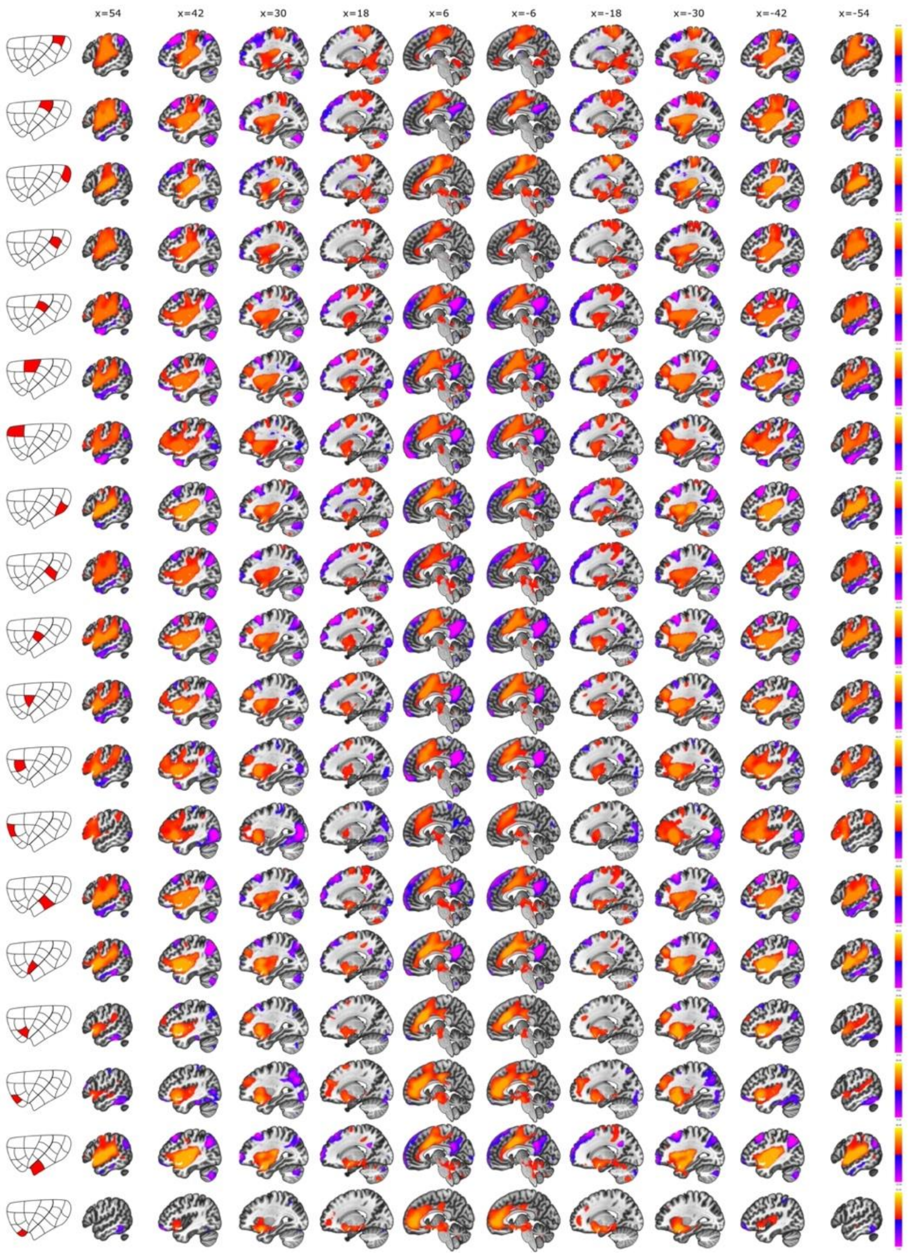

Figure S1.: Left connectivity profile of 19 insular subregions.

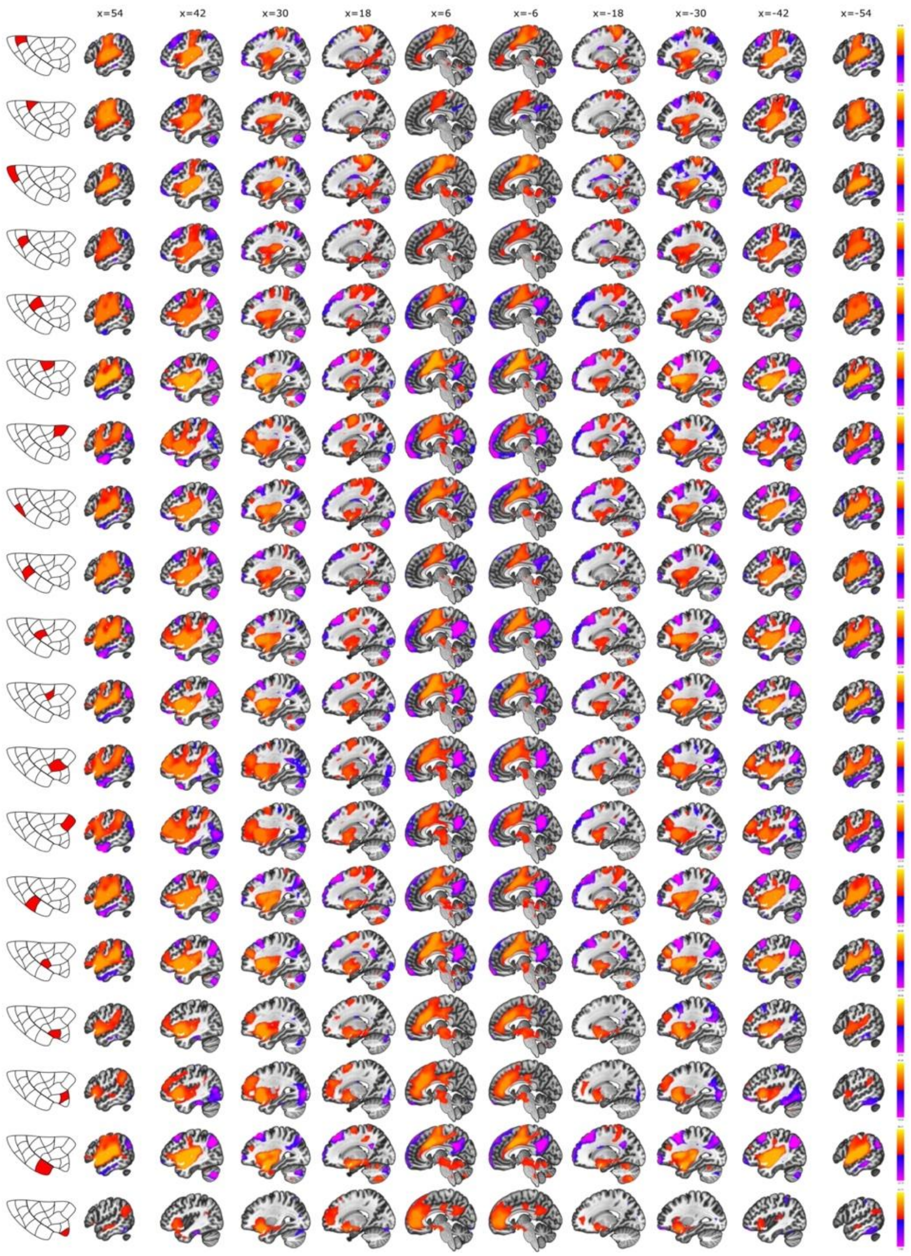

Figure S2.: Right connectivity profile of 19 insular subregions.

a.

dAI_left (K min. = 102; T (49) min. = 4.08)	
**Cluster (x, y, z)**	**Cluster Size**
-38 +14 -04	52803
+04 +62 -20	22557
+64 –04 -26	3508
-64 –10 –26	3176
-42 –74 -42	2365
-36 –34 +62	548
-04 –60 -50	845
+40 +34 –20	354
-34 –54 -36	294
-22 –72 -58	268
-42 +38 -20	257
+16 –74 -54	178
+02 +14 -16	159
+06 –36 +72	129
+34 –52 -36	120
-04 –42 -48	102

b.

dAI_right (K min = 98; T (49) min = 4.08)	
**Cluster (x, y, z)**	**Cluster size**
+34 +18 +04	51747
-44 –64 +28	18210
+06 +64 -14	12793
-36 –54 -34	1219
-34 –70 -02	655
-30 –80 -36	612
-36 –34 +62	610
+06 –58 -50	534
+40 +36 -22	270
-02 +12 -18	253
+16 –72 -52	154
-22 –18 -28	140

-24 –40 -16	138
+16 –70 +44	133
+72 –40 –06	120
-04 +24 +04	113
+36 –50 -34	98

c.

**dMI left (K min = 175; T (49) min = 4.05**	
**Cluster (x, y, z)**	**Cluster size**
-40 –02 +06	55148
+20 +32 +58	24109
+38 –70 -36	5620
+64 –10 –20	3012
-48 –60 +30	2778
-64 –10 –20	2600
-16 –72 -52	1319
-50 –62 -02	373
+00 –12 +16	183
+06 –56 -48	175

d.

**dMI right (K min = 227; T (49) min = 4.11**	
**Cluster (x, y, z)**	**Cluster size**
+38 +00 +06	48690
-36 +10 +48	14711
-06 –62 +40	7151
+24 –76 -40	2654
-32 –74 -38	1252
+68 –40 -10	1110
-60 –36 -10	1094
-14 –72 -52	564
+10 –72 -52	513
-34 +38 +22	433
+16 –62 -22	392
-20 -62 -26	328

+54 –56 –02	297
+06 -56 –52	274
-60 –64 -02	227

e.

**dPI left (K min = 89; T (49) min = 4.13**	
**Cluster (x, y, z)**	**Cluster size**
-38 –16 +14	52035
+32 +32 +54	10224
-34 –64 -44	4511
+38 –70 +54	2616
-48 –58 +50	1225
+10 –72 -50	1186
-26 +58 -10	944
+66 –32 -16	725
-58 –28 -16	145
-06 –74 +46	144
-64 –56 -12	96
+24 +46 –04	89

f.

**dPI right (K min = 549; T (49) min = 4.14**	
**Cluster (x, y, z)**	**Cluster size**
+38 –12 +10	50705
+38 +16 +60	14049
-32 –66 -42	2264
+38 –70 -32	1824
-30 +62 -06	1111
-16 –68 -54	968
-58 –32 -14	931
+58 –28 -18	549

g.

**vAI left (K min = 76; T (49) min = 4.24**	
**Cluster (x, y, z)**	**Cluster size**
-34 +12 -14	38627

-64 –46 -22	3116
+24 –84 -22	3085
-30 –70 +42	2231
+32 –58 +42	1738
+44 +50 -18	711
+36 +16 +62	442
-32 +12 +64	439
-46 +54 -02	385
+38 –64 +00	159
+36 –74 -56	101
-46 –06 -34	98
-26 –14 +34	76

h.

**vAI right (K min = 148; T (49) min = 3.50**	
**Cluster (x, y, z)**	**Cluster size**
+28 +14 -16	45074
-62 –44 -26	8058
+50 –34 -24	7457
-42 +50 -18	2064
-32 +12 +64	910
+28 –34 +06	548
+06 –64 -40	367
-10 +16 -28	148

i.

**vMI left (K min = 166; T (49) min = 4.20**	
**Cluster (x, y, z)**	**Cluster size**
-38 +04 -10	14154
+40 –06 -06	13950
-04 +18 +30	9135
-28 +22 +62	6728
+42 –72 +42	5633
-40 –66 +38	2763
+54 –30 -18	2121

+22 –82 –28	1471
-60 –28 –20	1390
-28 –80 –32	700
-16 –72 –50	294
+06 –90 –08	190
+12 –72 –50	176
-04 –62 -50	166

j.

vMI right (K min = 73; T (49) min = 4.18	
**Cluster (x, y, z)**	**Cluster size**
+38 +04 -08	32335
-38 –70 +38	10800
-02 +12 +34	7931
-38 +12 +46	5171
-04 +60 -22	2156
+38 –68 -40	1543
+64 –36 -10	1501
-58 –30 -18	887
-16 –72 -50	484
+14 –72 -54	361
-38 –72 –40	282
-04 –60 –50	258
-36 –54 -32	189
+40 +36 -22	118
-22 –02 +30	87
+04 –48 –18	87
-40 +12 –44	85
+34 –52 -32	73

k.

vPI left (K min = 153; T (49) min = 4.08	
**Cluster (x, y, z)**	**Cluster size**
-42 –08 -02	53533
+22 +32 +58	24665

+24 –80 -30	3357
+64 –14 -22	2292
-38 –68 -40	1557
+10 –72 -50	1047
-58 –64 +02	264
+06 –58 -48	172
+54 –56 +00	153

l.

**vPI right (K min = 102; T (49) min = 4.07**	
**Cluster (x, y, z)**	**Cluster size**
+40 –08 -04	55529
-40 –66 +38	25345
+38 –70 -38	2941
-58 –32 -16	1775
+64 –14 -2	1748
-42 –70 -38	1400
-34 +40 +22	523
-60 –68 +00	326
+32 +42 +24	233
+00 –10 +16	180
+04 -58 –48	136
+08 -102 -04	119
-26 +36 -16	102

m.

**Insula left vs right (K min = 64; T (49) min = 4.81**	
**Cluster (x, y, z)**	**Cluster size**
-38 +06 -10	4175
+36 +04 -06	2413
+28 +56 +10	415
+56 –48 +30	175
+38 +10 +46	137
+50 –62 +44	81
-14 –76 -28	79

+04 –46 -26	64

n.

**Dorsal anterior vs dorsal posterior left (K min = 82; T (49) min = 4.13**	
**Cluster (x, y, z)**	**Cluster size**
-36 +16 -04	27639
-36 –18 +12	24990
+02 –64 +28	12607
+02 +58 -22	3394
+58 –40 +52	2164
-56 –42 +42	1801
-34 –56 -38	977
-02 –60 –50	466
-02 +16 –16	369
+34 –56 –36	357
+14 –70 +48	310
+12 –34 +42	176
-26 –76 -58	107
+28 –80 -36	82

o.

**Dorsal anterior vs dorsal posterior right (K min = 146; T (49) min = 4.13**	
**Cluster (x, y, z)**	**Cluster size**
+38 –14 +10	35323
+34 +16 +00	25209
+00 +58 -18	4063
+54 –38 +40	2861
-36 –56 -38	1883
-56 –42 +36	1756
-22 –88 +00	688
+14 –34 +40	503
+02 –58 -50	503
+12 –68 +54	414
-08 –76 -26	215
+26 –78 -38	183

+34 –54 -38	164
-24 +44 –14	158
-44 +30 -16	146

p.

**Ventral anterior vs ventral posterior left (K min = 123; T (49) min = 3.50**	
**Cluster (x, y, z)**	**Cluster size**
-40 –14 +00	31235
-04 +50 +02	20982
-06 –48 +38	5957
-56 –30 -10	2400
+50 +06 -34	2120
-46 –58 -18	2055
-50 –58 +28	1710
+52 –66 -42	1469
+56 –56 -08	1362
-50 –66 -44	748
+14 –72 -48	742
-18 –70 -52	716
+34 +18 -14	600
+50 +42 +02	581
-44 +34 +12	365
+04 –42 -36	327
-16 –64 -24	186
-04 +08 -14	180
+30 –08 -44	160
+12 –22 +02	135
+18 –62 -24	123

q.

**Ventral anterior vs ventral posterior right (K min = 99; T (49) min = 4.14**	
**Cluster (x, y, z)**	**Cluster size**
+40 –08 -02	34360
+08 +48 +12	19525
-46 –64 +40	5754

-48 –62 -02	1459
+48 –58 +40	2182
+42 –66 -50	1170
-46 –68 -42	1147
+60 –24 -10	682
-12 –74 -52	561
+14 –74 -48	516
-56 –34 -12	419
+48 –04 -36	295
-28 +14 -14	186
+06 –58 -48	180
+52 +40 +02	171
+12 –22 +02	129
-52 –06 –30	120
+12 +18 +10	99

r.

**Dorsal vs ventral insula left (K min = 84; T (49) min = 4.29**	
**Cluster (x, y, z)**	**Cluster size**
-42 –02 +06	31337
+22 +46 +44	6636
+12 –52 +36	2433
-34 –80 -40	868
+38 +14 -16	702
+48 –58 +36	514
+28 –80 -42	293
+16 –72 -56	260
+50 –04 -42	200
-38 +36 +30	184
+62 –14 -16	177
+52 –46 -18	111
-44 –54 -12	103
-22 –72 –58	84

s.

Dorsal vs ventral insula right (K min = 91; T (49) min = 4.39	
**Cluster (x, y, z)**	**Cluster size**
+36 –14 +12	24531
+16 +58 +34	4823
+10 –52 +36	1084
+30 –82 -40	370
-28 –80 -38	322
-32 +12 -14	261
+06 +04 -12	123
+02 -22 +36	122
-20 -74 +22	118
+18 -64 +12	117
-52 –04 -36	111
-24 –20 -06	91

Table S2. Peak Insular subregions clusters coordinates (p< 0.001 FDR corrected at cluster level) for all 6 Rois in both hemispheres.

a. left dorsal anterior insula; **b.** right dorsal anterior insula; **c.** left dorsal middle anterior insula; **d.** right dorsal middle anterior insula; **e.** left dorsal posterior insula; **f.** right dorsal posterior insula; **g.** left ventral anterior insula; **h.** right ventral anterior insula; **i.** left ventral middle insula; **j.** right ventral middle insula; **k.** left ventral posterior insula; **l.** right ventral posterior insula; **m.** left vs right insula **n.** left dorsal anterior vs left dorsal posterior; **o.** right dorsal anterior vs right dorsal posterior; **p.** left ventral anterior vs left ventral posterior; **q.** right ventral anterior vs right ventral posterior; **r.** left dorsal vs left ventral insula; **s.** right dorsal vs right ventral insula.

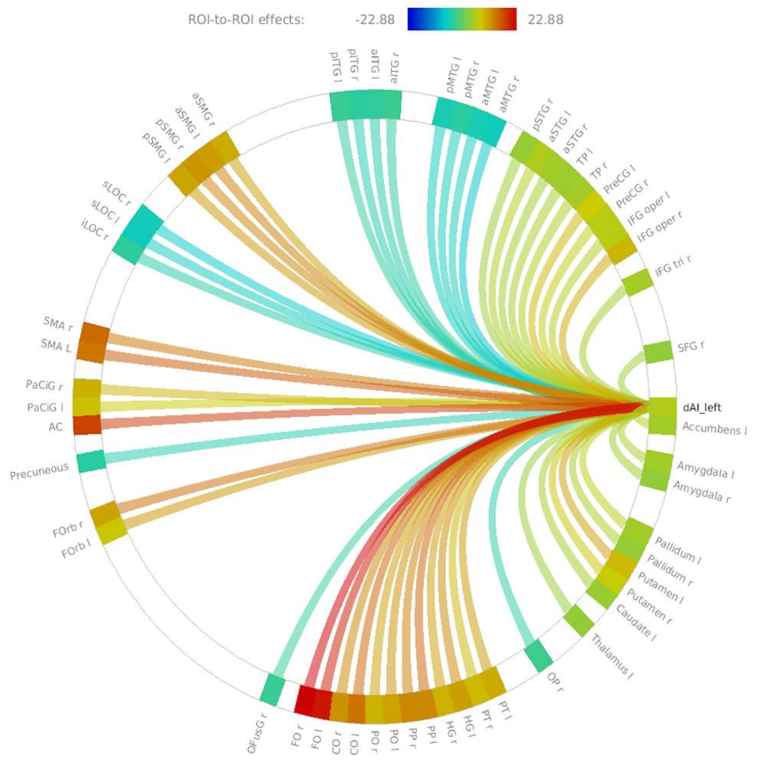

Figure S3. ROI to ROI connections of the left dAI presented as a connectome ring.

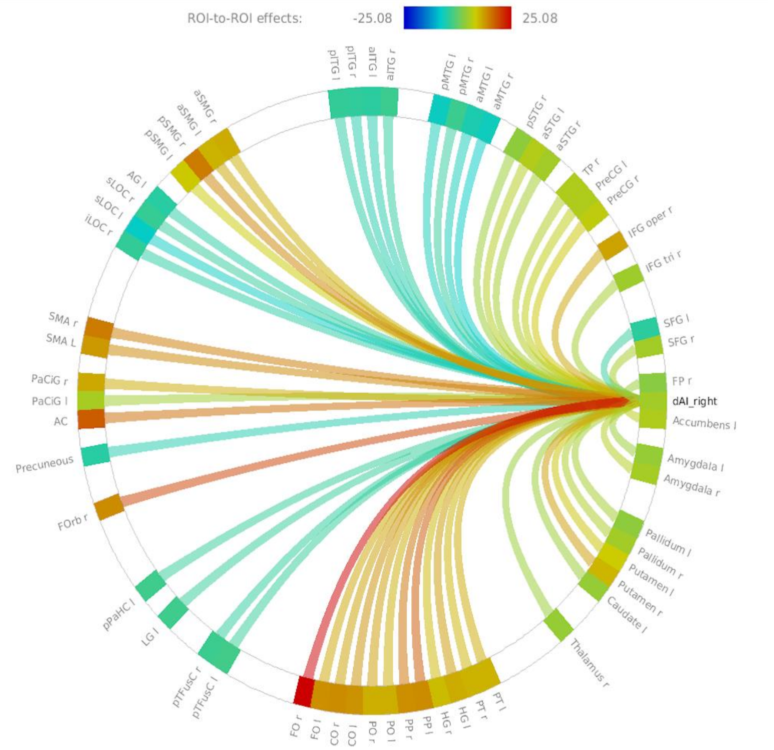

Figure S4. ROI to ROI connections of the left dMI presented as a connectome ring.

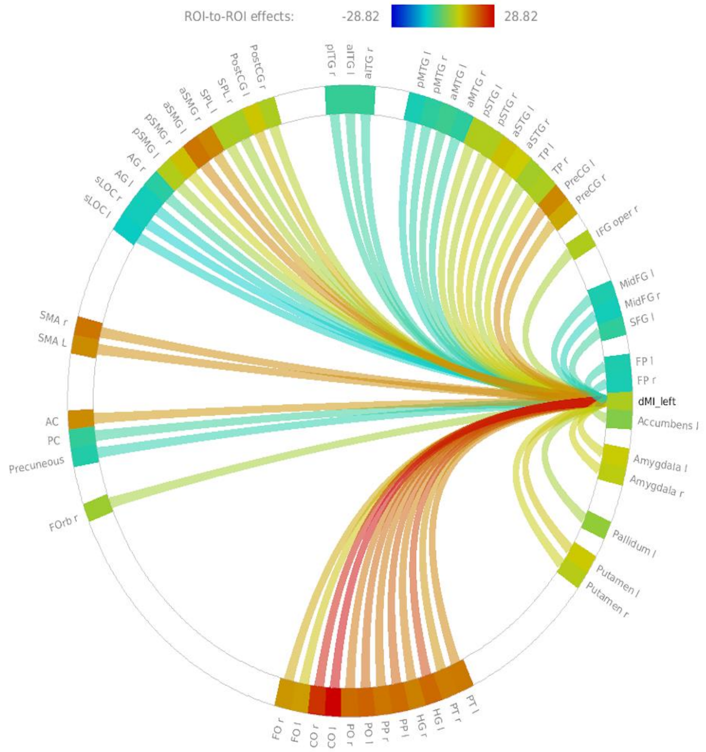

Figure S5. ROI to ROI connections of the right dMI presented as a connectome ring.

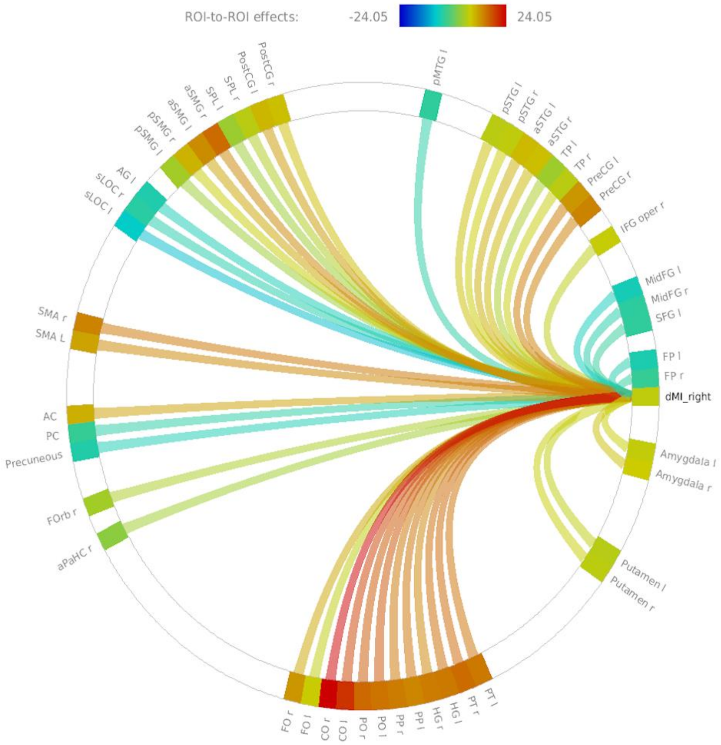

Figure S6. ROI to ROI connections of the right dMI presented as a connectome ring.

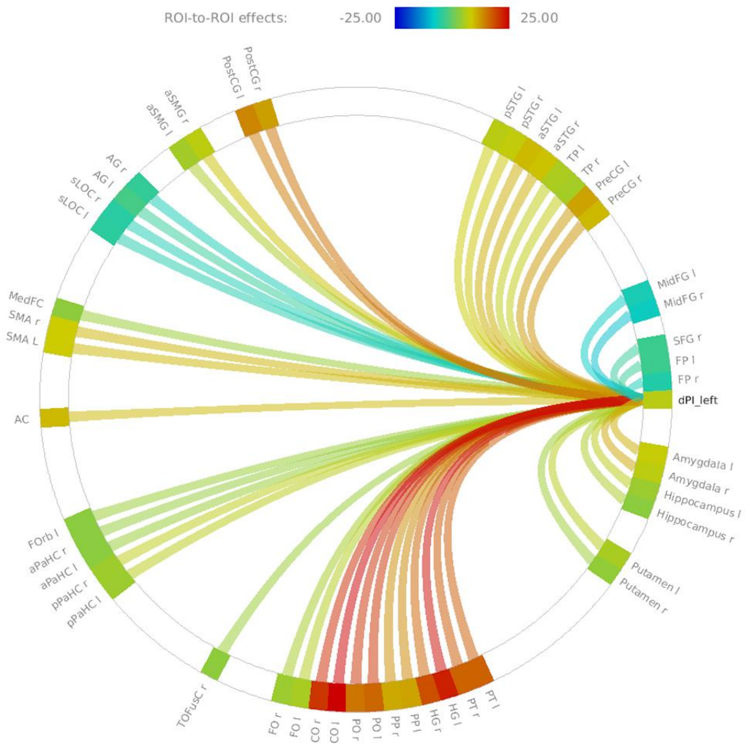

Figure S7. ROI to ROI connections of the left dPI presented as a connectome ring.

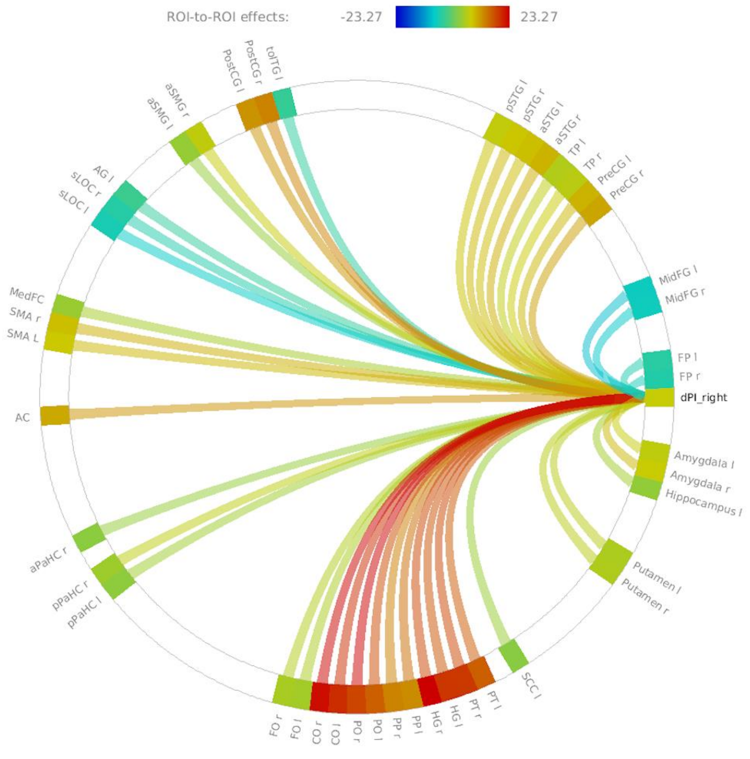

Figure S8. ROI to ROI connections of the right dPI presented as a connectome ring.

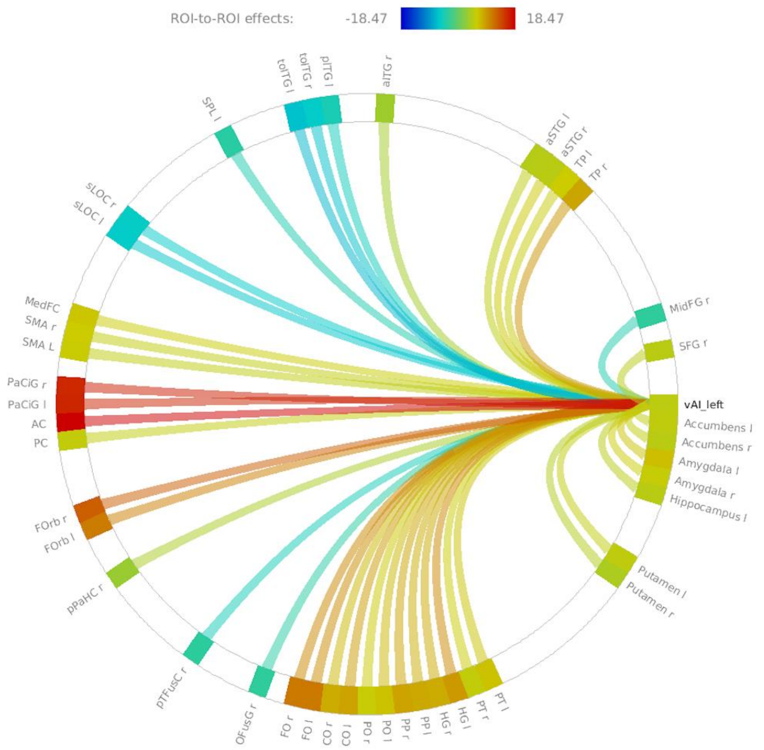

Figure S9. ROI to ROI connections of the left vAI presented as a connectome ring.

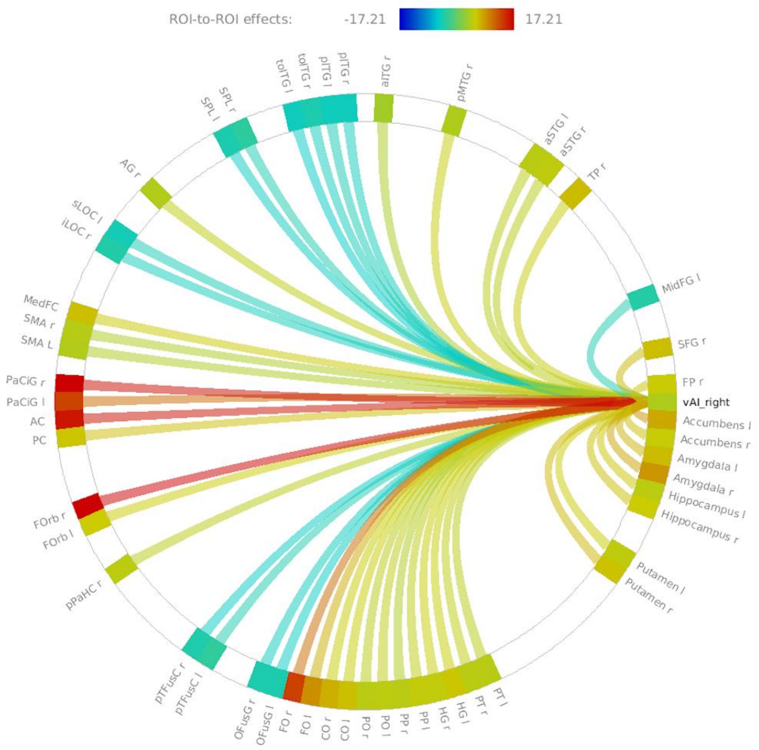

Figure S10. ROI to ROI connections of the right vAI presented as a connectome ring.

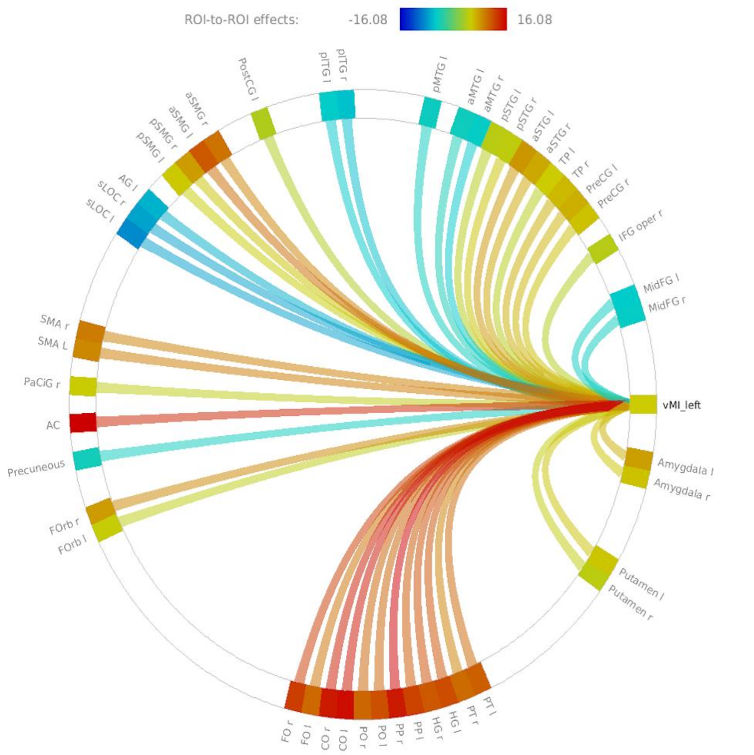

Figure S11. ROI to ROI connections of the left vMI presented as a connectome ring.

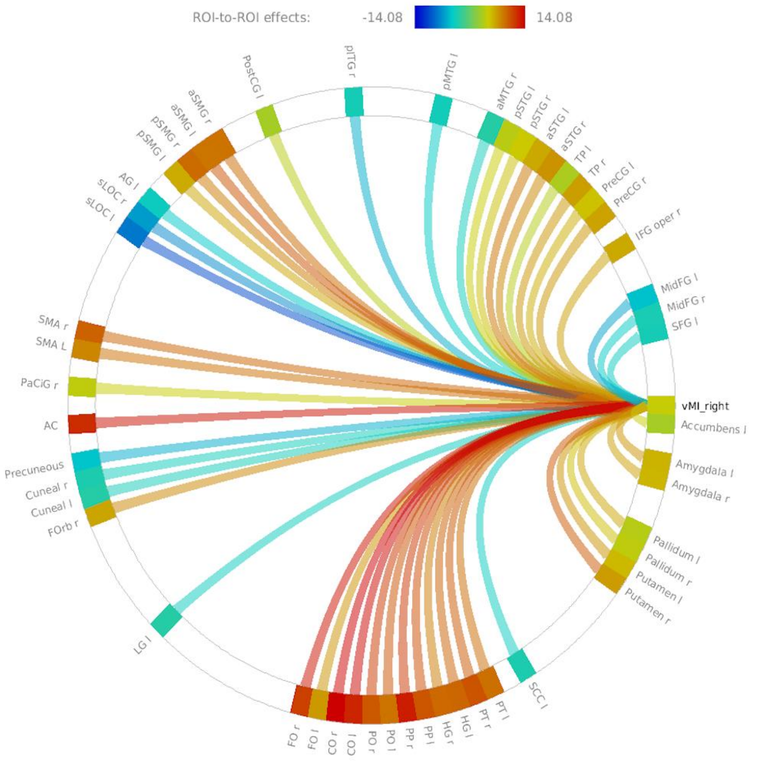

Figure S12. ROI to ROI connections of the right vMI presented as a connectome ring.
